# Supplementary material for: Fuel-Driven Catalytic Molecular Templating
Source: J Am Chem Soc. 2026 Jul 30;148(31):33027–38. doi: 10.1021/jacs.6c03709 (PMC13474929; doi:10.1021/jacs.6c03709)
Supplement: Supplementary file 1 [file ja6c03709_si_001.pdf]

Fuel-driven catalytic molecular templating

Merry Mitra, Rakesh Mukherjee, Križan Jurinović, and Thomas E. Ouldridge*

Department of Bioengineering and Centre for Engineering Biology, Imperial college London, London SW7 2AZ, UK

E-mail: t.ouldridge@imperial.ac.uk

Contents

SI.1 DNA strands and sequences **5**

SI.1.1 Description of DNA sequences for the first catalytic templating system 5

SI.1.2 DNA strands used to demonstrate orthogonality in the catalytic templating system . . . 7

SI.1.3 Description of strands used to demonstrate upstream control of catalytic templating . . . 8

SI.1.4 DNA strands used for experiments shown in figures . . . 10

SI.2 Experimental procedures **13**

SI.2.1 Annealing of complexes . . . 13

SI.2.2 Controls . . . 14

SI.2.3 Preparation of plate and reading with the plate reader . . . 15

SI.2.4 Fluorescence measurement settings . . . 17

SI.2.5 Protocols for each experiment . . . 18

SI.2.5.1 Test for reversibility . . . 20

SI.2.5.2 Optimization of catalytic templating turnover . . . 22

SI.2.5.3 Catalytic templating regulated by excess of fuel molecule . . . 24

SI.2.5.4 Catalytic templating with high monomer-to-template ratio . . . 25

SI.2.5.5 Reactions for orthogonal dimerization . . . 26

SI.2.5.6 Reactions with upstream gate circuitry . . . 31

SI.3 Data processing **36**

SI.3.1 General experimental context and common notation . . . 36

SI.3.1.1 Definitions . . . 37

SI.3.2 Positive control normalisation . . . 38

SI.3.2.1 Procedure . . . 38

SI.3.3 Conversion of AFU to concentrations for TMSD reactions (step 1) . . . 40

SI.3.3.1 Experimental context and signal model 40
SI.3.3.2 Concentration inference procedure (per trace) 41
SI.3.4 Conversion of AFU to concentrations for HMSD reactions (step 2) . . . 42
SI.3.4.1 Experimental context and signal model 42
SI.3.4.2 Concentration inference procedure 43
SI.3.5 Conversion of AFU to concentrations for internal TMSD reactions (step 3) 45
SI.3.5.1 Experimental context and signal model 45
SI.3.6 Calculation of a control-normalised readout in relative units 46
SI.3.6.1 Experimental context 46

SI.4 Kinetic model fitting 48

SI.4.1 Single step bimolecular reactions 48
SI.4.1.1 Mapping to assay-specific species. 49
SI.4.1.2 Fitting procedure 50
SI.4.1.3 Inference of a representative rate constant from replicate fits. . . 50
SI.4.2 Kinetic model fitting of catalytic turnover experiments 51
SI.4.2.1 Fitting procedure 52

SI.5 Supplementary results 55

SI.5.1 Individual steps 55
SI.5.1.1 Step 1 – toehold-mediated strand displacement 55
SI.5.1.2 Step 2 – handhold-mediated strand displacement 56
SI.5.1.3 Step 3 – internal toehold-mediated strand displacement 57
SI.5.1.4 Reversibility of step 1 61
SI.5.1.5 Reversibility of step 3 62
SI.5.2 Optimization of catalytic templating turnover 63
SI.5.2.1 Optimization of the toehold and the handhold lengths 63

SI.5.3 Catalytic templating regulated by excess of fuel molecule 65
SI.5.4 Catalytic templating with high monomer-to-template ratio 66
SI.5.5 Reactions for orthogonal dimerization 67
SI.5.5.1 Full reaction for orthogonal set 2 67
SI.5.5.2 Dimerization reactions of each pair of monomers triggered with four different templates 68
SI.5.5.3 Specificity of the templates in a pool of monomers 69
SI.5.5.4 Gel showing non-reactivity of second monomer and fuel prior reaction 71
SI.5.5.5 Catalytic templating reactions with variable concentrations of inputs 72
SI.5.5.6 Schematic of a trimerization reaction network design following the proposed mechanism 73

SI.1 DNA strands and sequences

Each strand used in the the reported work is described in Tables S1, S3 and S5. All sequences used are listed in Tables S2, S4 and S6.

SI.1.1 Description of DNA sequences for the first catalytic templating system

All strand sequences are written from 5'to 3'. Poly T linkers are used to connect the strands to fluorophores, or to produce different migration rates in PAGE gels, where necessary. Sequences are coloured by domains using the following scheme:

Lilac: Toehold and its complement in monomer 1 and templates.

Red: Branch migration domain and its complement that binds monomer 1 to the fuel.

Black: Branch migration domain and its complement that binds monomer 1 to monomer 2.

Blue: internal toehold in monomer 2 and its complement in the fuel.

Yellow: handold contained in monomer 2 and its complement in the templates.

Grey and bold: Sequence mismatches whose repair drive the dimerization reaction.

Table S1. Description of strands used for the first catalytic templating system.

**Strands**	**Description**	**Variants with labelling**
S_1	First monomer of the first set. Contains the toehold and is initially blocked.	Two variants:<br><br>a) fluorophore on 3' end;<br>b) fluorophore on 5' end.
R_1	Second monomer of the first set. Contains the handhold and internal toehold.	Two variants:<br><br>a) fluorophore on 5' end;<br>b) unlabelled.
L	Blocker that initially blocks monomer S_1.	Quencher on 5' end.
T_{11} (also called $T^{8\text{-}7}$)	Template T_{11} with handhold length 8 nt and toehold length 7 nt and dimerizes S_1 and R_1.	Two variants:<br><br>a) unlabelled;<br>b) quencher on 3' end.
$T^{p\text{-}q}$	To test variants of toehold and handhold lengths, we used templates $T^{p\text{-}q}$, where 'p' is the length of the handhold, and 'q' is the length of toehold.	Unlabelled.
D^j	Fuel with internal toehold length 'j'.	Unlabelled.

Table S2. DNA sequences used for the first catalytic templating system.

**Strands**	**DNA Sequences**
S_1(a)	CCCTTAACCTACCCTCACATCTCCACCTCAACCTAACTCCTAACTACTACTGGCC TTT ATTO 488
S_1(b)	ATTO 488 TTT CCCTTAACCTACCCTCACATCTCCACCTCAACCTAACTCCTAACTACTACTGGCC
R_1(a)	Alexa 546 TTT GGCCAGTAGTAGTTAGGAGTTAGGTCCATCATCCATCAAATTCAC
R_1(b)	GGCCAGTAGTAGTTAGGAGTTAGGTCCATCATCCATCAAATTCAC
L	Iowa Black FQ TTT GGCCAGTACTAGTTACGAGTTACGTTAGGTGCAGATGTCAGGGTAGG
$T_{11}/T^{8\text{-}7}$(a)	GAATTTGAGCCAGTACTAGTTACGAGTTACGTTAGGTGCAGATGTCAGGGTAGGTTAAGGG
$T_{11}/T^{8\text{-}7}$(b)	GAATTTGAGCCAGTACTAGTTACGAGTTACGTTAGGTGCAGATGTCAGGGTAGGTTAAGGG TTT Iowa Black FQ
$T^{9\text{-}7}$	TGAATTTGAGCCAGTACTAGTTACGAGTTACGTTAGGTGCAGATGTCAGGGTAGGTTAAGGG
$T^{9\text{-}6}$	TGAATTTGAGCCAGTACTAGTTACGAGTTACGTTAGGTGCAGATGTCAGGGTAGGTTAAGG
$T^{8\text{-}6}$	GAATTTGAGCCAGTACTAGTTACGAGTTACGTTAGGTGCAGATGTCAGGGTAGGTTAAGG
$T^{7\text{-}7}$	AATTTGAGCCAGTACTAGTTACGAGTTACGTTAGGTGCAGATGTCAGGGTAGGTTAAGGG
$T^{7\text{-}6}$	AATTTGAGCCAGTACTAGTTACGAGTTACGTTAGGTGCAGATGTCAGGGTAGGTTAAGG
$T^{0\text{-}7}$	GCCAGTACTAGTTACGAGTTACGTTAGGTGCAGATGTCAGGGTAGGTTAAGGG
D^{10}	TGGATGATGGTAGGTGGAGATGTGAGGGTAGG
D^7	ATGATGGTAGGTGGAGATGTGAGGGTAGG
D^6	TGATGGTAGGTGGAGATGTGAGGGTAGG
D^5	GATGGTAGGTGGAGATGTGAGGGTAGG

SI.1.2 DNA strands used to demonstrate orthogonality in the catalytic templating system

All the templates used in these reactions have handhold of length 8 nt and toehold 7 nt. These strands were used along with S_1, R_1, L, T_{11}, D^7, as described in Table S1. Sequences are coloured using the same colour scheme.

Table S3. Strands used to demonstrate orthogonality in the catalytic templating system.

Strands	Description	Variants with labelling
S_2	First monomer of the second. Contains a toehold orthogonal to that of S_1, but the same branch migration domains.	Two variants:<br>a) fluorophore on 3' end;<br>b) fluorophore on 5' end.
R_2	Second monomer of the second set. Contains a handhold orthogonal to that of R_1, but the same internal toehold and branch migration domain.	Two variants:<br>a) unlabelled with 3T on 5' end;<br>b) unlabelled with 7T each on both the ends.
T_{22}	Template T_{22} that dimerizes S_2 and R_2	Two variants:<br>a) unlabelled;<br>b) quencher on 3' end.
T_{12}	Template T_{12} that dimerizes S_1 and R_2	Two variants:<br>a) unlabelled;<br>b) quencher on 3' end.
T_{21}	Template T_{21} that dimerizes S_2 and R_1	Two variants:<br>a) unlabelled;<br>b) quencher on 3' end.

Table S4. DNA sequences to demonstrate orthogonal catalytic templating.

**Strands**	**DNA Sequences**
S_2(a)	CGCTCTACCTACCCTCACATCTCCACCTCAACCTAACTCCTAACTACTACTGGCC TTT Alexa 546
S_2(b)	Alexa 546 TTT CGCTCTACCTACCCTCACATCTCCACCTCAACCTAACTCCTAACTACTACTGGCC
R_2(a)	TTT GGCCAGTAGTAGTTAGGAGTTAGGTCCATCATCCATCCATCTCAA
R_2(b)	TTTTTTT GGCCAGTAGTAGTTAGGAGTTAGGTCCATCATCCATCCATCTCAA TTTTTTT
T_{22}(a)	GAGATGGAGCCAGTACTAGTTACGAGTTACGTTAGGTGCAGATGTCAGGGTAGGTAGAGCG
T_{22}(b)	GAGATGGAGCCAGTACTAGTTACGAGTTACGTTAGGTGCAGATGTCAGGGTAGGTAGAGCG TTT Iowa Black FQ
T_{12}(a)	GAGATGGAGCCAGTACTAGTTACGAGTTACGTTAGGTGCAGATGTCAGGGTAGGTTAAGGG
T_{12}(b)	GAGATGGAGCCAGTACTAGTTACGAGTTACGTTAGGTGCAGATGTCAGGGTAGGTTAAGGG TTT Iowa Black FQ
T_{21}(a)	GAATTTGAGCCAGTACTAGTTACGAGTTACGTTAGGTGCAGATGTCAGGGTAGGTAGAGCG
T_{21}(b)	GAATTTGAGCCAGTACTAGTTACGAGTTACGTTAGGTGCAGATGTCAGGGTAGGTAGAGCG TTT Iowa Black FQ

SI.1.3 Description of strands used to demonstrate upstream control of catalytic templating

The strands described in Table S5 were used along with S_1(a), R_1(a), L and T_{11}(a), as described in Table S1.

Sequences in Table S6 are coloured by domain using the following colours:

Teal: extension of fuel Dn that allows it to bind with with X or XE, sand its complement. Branch migration domain of inputs I1, $I1_M$, Z and $\bar{I1}_M$.

Blue: internal toehold in fuel Dn.

Red: Branch migration domain of fuel Dn that allows it to bind to S_1.

Orange: toehold and its complement in X, XE, and inputs I1, $I1_M$, $\bar{I1}_M$.

Lavender: branch migration domain and its complement in input I2 and XE.

Navy: toehold and its complement in Input I2 and XE

Grey and bold: mismatches

Table S5. Description of strands used for the upstream gate circuitry.

**Strands**	**Description**	**Used in gated systems**	**Variants with labelling**
Dn	An extended fuel strand with internal toehold length 7 nt which is initially bound to X or XE	all	unlabelled
X	Fuel Dn is bound to strand X	Single-input, catalytic, NOT	quencher on 5' end
I1	Input strand	Single-input, 2-input AND	fluorophore on 3' end
E	Initially bound to XE strand, which detaches to open up a toehold for the input I2	2-input AND	fluorophore on 5' end
XE	Fuel Dn and strand E is initially bound to XE	2-input AND	quencher on 5' end
I2	Input strand	2-input AND	unlabelled
$I1_M$	Input strand, which is similar to input I1, but with one nt change (C instead of G at 24th position) to retain a mismatch in the second step of the reaction	catalytic, NOT	fluorophore on 3' end
Z	Ancillary strand	catalytic, NOT	unlabelled
$\bar{I1}_M$	Input strand – fully complementary to $I1_M$	NOT	unlabelled

Table S6. DNA sequences used for the upstream gate circuitry.

**Strands**	**DNA Sequences**
Dn	GGCGATTTCAGTGGAGCTTTAGTTGATGATGGTAGGTGGAGATGTGAGGGTAGG
X	Iowa Black FQ TTT CCATCATCAACTAAACCTCCACTCAAATCGCCAAATAGA
I1	TCTATTTGGCGATTTGAGTGGAGGTTTAGTTG TTT ATTO 590
E	ATTO 425 TTT GTAGTTATCATTTGGCCAGGTTAGTTCTATTT
XE	Iowa Black FQ TTT<br>CCATCATCAACTAAACCTCCACTCAAATCGCCAAATAGAACTAACCTCGCCAAATCATAACTACACTCACC
I2	GGTGAGTGTAGTTATGATTTGGCGAGGTTAGT
$I1_M$	TCTATTTGGCGATTTGAGTGGAGCTTTAGTTG TTT ATTO 590
Z	GGCGATTTGAGTGGAGGTTTAGTTGATGATGG
$\bar{I1}_M$	CAACTAAAGCTCCACTCAAATCGCCAAATAGA

SI.1.4 DNA strands used for experiments shown in figures

The strands used in the reported figures of both the main text and the supplementary information are listed in Tables S7 and S8.

Table S7. DNA strands used in experiments reported in the main text.

**Figure numbers**	**Strands used**
2a	S_1(a), L, T_{11}(a)
2b	S_1(a), T_{11}(a), R_1(a)
2c	S_1(b), T_{11}(b), R_1(b), D^7
3a	S_1(a), L, R_1(b), D^5, T_{11}(a)
3b	S_1(a), L, R_1(b), D^6, T_{11}(a)
3c	S_1(a), L, R_1(b), D^7, T_{11}(a)
3d	S_1(a), L, R_1(b), D^{10}, T_{11}(a)
3e	S_1(a), L, R_1(b), D^6, T_{11}(a)
3f	S_1(a), L, R_1(b), D^7, T_{11}(a)
4a	S_1(a), L, R_1(b), D^7, T_{11}(a), T_{12}(a), T_{21}(a), T_{22}(a)
4b	S_1(a), L, R_1(b), S_2(a), R_2(a), D^7, T_{11}(a), T_{12}(a), T_{21}(a), T_{22}(a)
4c	S_1(a), L, R_1(b), S_2(a), R_2(a), D^7, T_{11}(a), T_{12}(a), T_{21}(a), T_{22}(a)
4e	S_1(a), S_2(a), L, R_1(b), R_2(b), D^7, T_{11}(a), T_{12}(a), T_{21}(a), T_{22}(a)
6a	Dn, X, I1, S_1(a), L, R_1(a), T_{11}(a)
6b	Dn, E, XE, I2, I1, S_1(a), L, R_1(a), T_{11}(a)
6c	Dn, X, $I1_M$, Z, S_1(a), L, R_1(a), T_{11}(a)
6d	Dn, X, $I1_M$, Z, $I\bar{1}_M$, S_1(a), L, R_1(a), T_{11}(a)
6e	Dn, X, $I1_M$, Z, S_1(a), L, R_1(a), T_{11}(a)

Table S8. DNA strands used in experiments reported in supporting figures.

**Figure numbers**	**Strands used**
S2a	S_1(a), L, $T^{8\text{-}6}$
S2b	S_1(a), L, $T^{8\text{-}7}$
S3a	S_1(a), $T^{0\text{-}7}$, R_1(a)
S3b	S_1(a), $T^{7\text{-}7}$, R_1(a)

S3c	S_1(a), $T^{8\text{-}7}$(a), R_1(a)
S3d	S_1(a), $T^{9\text{-}7}$, R_1(a)
S4a	S_1(b), T_{11}(b), R_1(b), D^5
S4b	S_1(b), T_{11}(b), R_1(b), D^6
S4c	S_1(b), T_{11}(b), R_1(b), D^7
S4d	S_1(b), T_{11}(b), R_1(b), D^{10}
S5	S_1(a), T_{11}(a), L
S6a	S_1(b), R_1(b), D^5, T_{11}(b)
S6b	S_1(b), R_1(b), D^6, T_{11}(b)
S6c	S_1(b), R_1(b), D^7, T_{11}(b)
S6d	S_1(b), R_1(b), D^{10}, T_{11}(b)
S7a	S_1(b), R_1(b), D^7, T_{11}(b)
S7b	S_1(b), R_2(a), D^7, T_{12}(b)
S7c	S_2(b), R_1(b), D^7, T_{21}(b)
S7d	S_2(b), R_2(a), D^7, T_{22}(b)
S8a	S_1(a), L, R_1(a), D^7, $T^{9\text{-}7}$
S8b	S_1(a), L, R_1(a), D^7, $T^{8\text{-}7}$
S8c	S_1(a), L, R_1(a), D^7, $T^{7\text{-}7}$
S8d	S_1(a), L, R_1(a), D^7, $T^{9\text{-}6}$
S8e	S_1(a), L, R_1(a), D^7, $T^{8\text{-}6}$
S8f	S_1(a), L, R_1(a), D^7, $T^{7\text{-}6}$
S9a	S_1(a), L, R_1(b), D^7, T_{11}(a)
S9b	S_1(a), L, R_1(b), D^6, T_{11}(a)
S10a-b	S_1(a), L, R_1(b), D^7, T_{11}(a)
S11	S_2(a), L, R_2(a), D^7, T_{22}(a)
S12a	S_1(a), L, R_2(a), D^7, T_{11}(a), T_{12}(a), T_{21}(a), T_{22}(a)
S12b	S_2(a), L, R_1(b), D^7, T_{11}(a), T_{12}(a), T_{21}(a), T_{22}(a)
S12c	S_2(a), L, R_2(a), D^7, T_{11}(a), T_{12}(a), T_{21}(a), T_{22}(a)
S13	S_1(a), S_2(a), L, R_1(b), R_2(b), D^7, T_{11}(a), T_{12}(a), T_{21}(a), T_{22}(a)
S14	R_1(a), D^{10}, D^7, D^6
S15a	Dn, X, I1, S_1(a), L, R_1(a), T_{11}(a)

S15b	Dn, X, $I1_M$, Z, S_1(a), L, R_1(a), T_{11}(a)

SI.2 Experimental procedures

SI.2.1 Annealing of complexes

Annealing was performed according to the protocol reported in the methods section of the manuscript. Working solutions were prepared with a final concentration as tabulated in Tables S9 – S10. Excesses of one strand were used to ensure all complementary partners were bound.

Table S9. Concentrations of DNA strands in working solutions of annealed complexes used to demonstrate catalytic templating in both the orthogonal sets.

**Complexes**	**Strand names**	**Strand annotations**	**Final conc (nM)**	**Description**
Reactant S_1-L/S_2-L	Monomer 1	S_1(a)/S_2(a)	200	
	Blocker	L	400	100% excess
Control S_1-T	Monomer 1	S_1(a)	200	
	Template	T_{11}(a)/$T^{p\text{-}q}$	240	20% excess
Reactant S_1-T	Monomer 1	S_1(a)	200	
	Template	T_{11}(a)/$T^{p\text{-}q}$	300	50% excess
Control S_1-R_1-T	Monomer 1	S_1(a)	200	
	Monomer 2	R_1(a)	240	20% excess
	Template	T_{11}(a)/$T^{p\text{-}q}$	240	20% excess
Reactant S_1-R_1-T	Monomer 1	S_1(b)	200	
	Monomer 2	R_1(b)	300	50% excess
	Template	T_{11}(b)	300	50% excess
Controls S_1-R_1-D, S_1-R_2-D, S_2-R_1-D, S_2-R_2-D	Monomer 1	S_1(a)/S_1(b)/ S_2(a)/S_2(b)	200	
	Monomer 2	R_1(a)/R_1(b)/R_2	240	20% excess
	Fuel	D^j	240	20% excess

Table S10. Concentrations of DNA strands in working solutions of annealed complexes used to demonstrate catalytic templating coupled with the upstream DNA gate circuitry.

**Strand names**	**Strand annotations**	**Final conc (nM)**	**Description**
Dn-X	Dn	200	
	X	220	10% excess
E-Dn-XE	Dn	200	
	E	240	20% excess
	XE	220	10% excess
Control S_1-R_1-Dn	S_1(a)	200	
	R_1(a)	240	20% excess
	Dn	240	20% excess

SI.2.2 Controls

In all experiments, we used four different concentrations for the positive control (run-time control), except the specificity experiments shown in Table S23 and S24 where we have used two different concentrations for each set of reactions; at least one negative control (where the triggering agent/(s) is/are absent) and a blank consisting of 1x TAE buffer (working buffer). The controls were subjected to identical volume changes and environmental conditions as those experienced by the samples during the kinetic measurements. These controls enabled correction for fluorescence changes arising from environmental variations, and the procedure for data correction using the control signals is described in section SI.3.2.

SI.2.3 Preparation of plate and reading with the plate reader

At the start of the experiment, all the reactants (except the triggering agent/s) were added at the desired concentrations and volumes to the experimental buffer (1M NaCl in 1x TAE) to obtain a total reaction volume of 150 µL. The experimental concentrations reported in both the main text and supplementary information represent the final concentrations in a total volume of 200 µL, accounting for the additional 50 µL that was introduced during the injection step. All the different fluorescence measurements in the different experiments followed a similar structure, outlined below:

Measurement 1. Initial fluorescence: We took an initial measurement (baseline) with incubation at 25 °C to gather at least 20 data points after the fluorescence signals of the positive controls became stable. This approach allows us to minimize the uncertainties in concentration that may result from factors such as temperature variations, pipetting errors, and other experimental inconsistencies.

Measurement 2. Injection of triggering agent and kinetic measurement: For the majority of experiments, 50 µL of the triggering reactant was injected by the plate reader, bringing the total reaction volume to 200 µL. For all but the fastest reactions, the plate was then completely sealed to prevent evaporation, after a short period in which the initial dynamics was monitored carefully. The fluorescence signal was then monitored for long enough to reach steady state for all but the slowest of reactions. In some experiments with slower kinetics in which multiple different triggers were added to the same plate, the triggering was instead performed by hand, the plate sealed, and added to the plate reader. The endpoint fluorescence of measurement 2 can be used to infer concentrations of certain initial reactants (assuming irreversibility and reaction completion).

Measurement 3. Saturation: To finish the assay, experiments were often saturated with an excess (which must not be any fluorophore-containing species) of a non-fluorescent reagent. This saturation allowed an estimate of the initial concentration of reactants where this in-

formation is required.

All the procedures mentioned above are illustrated in Figure S1 and the measurements are annotated as m1-m3.

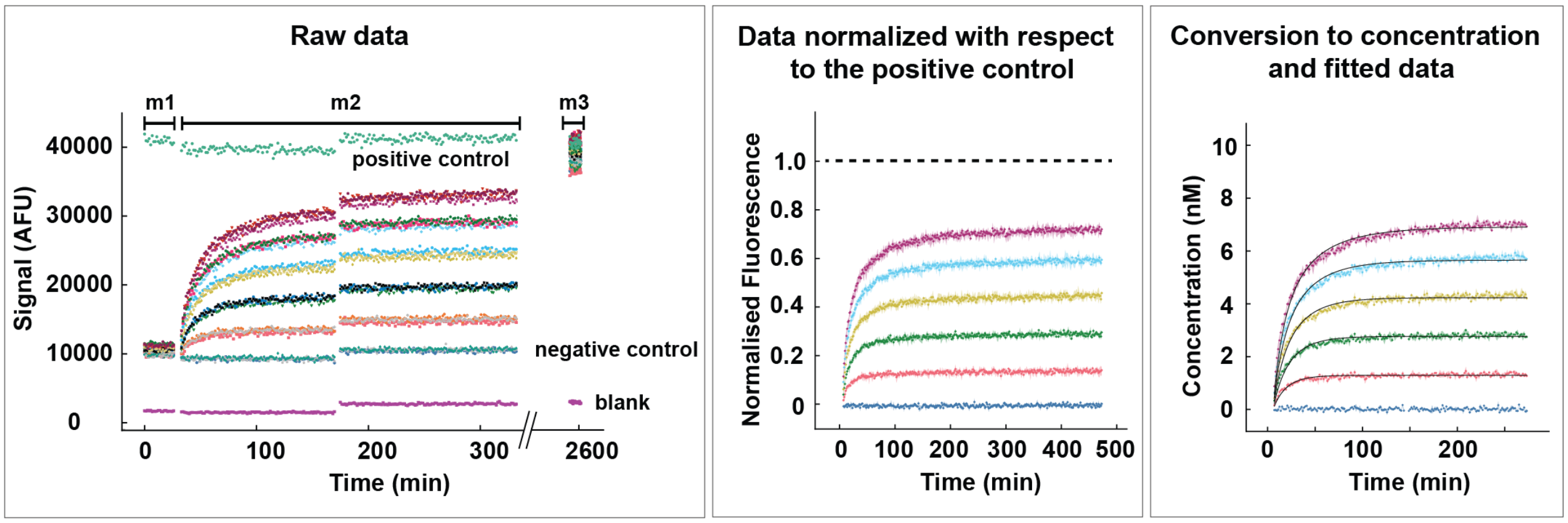

Figure S1. Demonstration of a complete set of measurements for a general reaction using a Microplate reader and the processing of data. In the left panel, 'm1' represents the baseline of the experiments, considered as 0 nM of reacted species, before the injection of the triggering agent. At the start of 'm2', varying amounts of the triggering reactant were added and the reaction was then monitored. The plate was sealed at around 175 minutes, and then monitored for a further few hours to complete m2. Afterwards, 'm3' was taken where the reaction mixtures were saturated with a large excess of the non-fluorescent reactant, sufficient enough so that the fluorophore-labelled species react completely. The negative controls consists of all the reactants, except the triggering agent. Although 4 postive controls at different concentrations were used in experiments, we plot only one here for ease of interpretation. After data processing (section SI.3) and kinetic fitting (section SI.4) the figures (middle and right) in the subsequent panels are obtained.

SI.2.4 Fluorescence measurement settings

The kinetic measurements were obtained by monitoring the fluorescence of fluorophore-labelled strands. The experimental settings for each fluorophore are reported in Table S11.

Table S11. Settings for monitoring of fluorescence

**Fluorophores**	**Excitation**	**Dichroic filter**	**Emission**	**Gain**	**Gain for high conc samples**
ATTO 488	488–14	504.8	519–9	2500	2200
Alexa Fluor 546	540–20	562.5	590–30	1800	1400
FRET: ATTO 488 – Alexa Fluor 546	488–10	534	590–30	2000	1600
ATTO 425	430–15	461.2	490–40	2400	–
ATTO 590	575–30	602	634–40	2000	–

The specific procedures for each type of experiment are discussed and collected in Tables in the following subsections.

SI.2.5 Protocols for each experiment

In Tables S12 - S30, each **concentration** column represents a single reaction well, containing the reactants – listed row by row – in the intended concentrations in 200 µL volume.

A. Step 1 – toehold-mediated strand displacement. Increasing concentrations (0–10 nM) of the template $T^{p\text{-}q}$ were added to wells containing 10 nM of pre-annealed, blocked monomer S_1(a)-L. After the signal reached a plateau, all reaction wells were treated with large excess of $T^{p\text{-}q}$ to ensure complete consumption of any unreacted monomer in the reaction mixture. The reaction conditions are tabulated in Table S12.

Table S12. Reaction protocol for step 1 for experiments shown in Figures 2a in the manuscript and S2.

**Reactants**	**Concentrations in nM**					
S_1-L	10	10	10	10	10	10
$T^{p\text{-}q}$ (added by injector)	0	2	4	6	8	10

**Positive Control**	**Concentrations in nM**			
S_1-$T^{p\text{-}q}$	5	10	15	20

B. Step 2 – handhold-mediated strand displacement. To investigate the second step, monomer S_1(a) and template $T^{p\text{-}q}$(a) were pre-annealed. Variable concentrations (0–10 nM) of R_1(a) were added to wells containing 10 nM of pre-annealed, template bound monomer S_1(a). The reaction conditions are tabulated in Table S13.

Table S13. Reaction protocol for step 2 for experiments shown in Figures 2b in the manuscript and S3.

**Reactants**	**Concentrations in nM**					
S_1-$T^{p\text{-}q}$	10	10	10	10	10	10
R_1 (added by injector)	0	2	4	6	8	10

**Positive Control**	**Concentrations in nM**			
S_1-R_1-$T^{p\text{-}q}$	5	10	15	20

C. Step 3 – internal toehold-mediated strand displacement. 10 nM of the pre-annealed complex S_1(b)-R_1(b)-T_{11}(b) were added to the varying concentrations (0–10 nM) of the fuels D^j. After the signal reached a plateau, we saturated the reaction mixtures with excess of D^j in respective wells to ensure complete consumption of any unreacted complex S_1-R_1-T_{11} in the reaction mixture. The reaction conditions are tabulated in Table S14.

Table S14. Reaction protocol for step 3 for experiments shown in Figures 2c in the manuscript and S4.

**Reactants**	**Concentrations in nM**					
D^j	0	2	4	6	8	10
S_1-R_1-T_{11} (added by injector)	10	10	10	10	10	10

**Positive Control**	**Concentrations in nM**			
S_1-R_1-D^j	5	10	15	20

SI.2.5.1 Test for reversibility

To check the thermodynamic drive associated with the templating reaction, we examined the irreversibility of product formation for step 1 and step 3 in the catalytic cycle.

A. Reversibility of step 1. We reacted 10 nM of S_1(a)-T_{11}(a) with variable concentrations (0-10 nM) of the blocker L. The reaction conditions are tabulated in Table S15.

Table S15. Reaction protocol for the reversibility test for step 1 for experiments shown in Figure S5.

**Reactants**	**Concentrations in nM**					
S_1-T_{11}	10	10	10	10	10	10
L (added by injector)	0	2	4	6	8	10

**Positive Control**	**Concentrations in nM**			
S_1-L	5	10	15	20

B. Reversibility of step 3. We first reacted 10 nM of pre-annealed complex S_1(b)-R_1(b)-D^j (j = 5, 6, 7 and 10) with variable concentrations (0-10 nM) of T_{11}(b). We then repeated the exercise using all 4 orthogonal templates and their target products for D^7. For these reactions, pre-annealed complexes S_1(b)-R_1(b)-D, S_1(b)-R_2(a)-D, S_2(b)-R_1(b)-D and S_2(b)-R_2(a)-D were individually reacted to T_{11}(b), T_{12}(b), T_{21}(b) and T_{22}(b) respectively. The reaction conditions are tabulated in Table S16.

Table S16. Reaction protocol for the reversibility test for step 3 for experiments shown in Figures S6 and S7.

**Reactants**	**Concentrations in nM**					
S_k-R_l-D^j	10	10	10	10	10	10
T_{kl} (added by injector)	0	2	4	6	8	10

**Positive Control**	**Concentrations in nM**			
S_k-R_l-T_{kl}	5	10	15	20

SI.2.5.2 Optimization of catalytic templating turnover

A. Optimization of the toehold and the handhold lengths. Varying concentrations (0–5 nM) of the template $T^{p\text{-}q}$ were added to wells containing 10 nM of each of pre-annealed complex S_1(a)-L, monomer R_1(a), and fuel D^7, considering 6-7 for q and 7-9 for p. The reaction procedure is tabulated in Table S17.

Table S17. Reaction protocol for catalytic templating with templates of varying toehold and handhold lengths for experiments shown in Figure S8.

**Reactants**	**Concentrations in nM**				
S_1-L	10	10	10	10	10
R_1	10	10	10	10	10
D^7	10	10	10	10	10
$T^{p\text{-}q}$ (added by injector)	0	2	3	4	5

**Positive Control**	**Concentrations in nM**			
S_1-R_1-D^7	5	10	15	20

B. Optimization of the internal toehold length. We added varying concentrations of template $T^{8\text{-}7}$ (also known as T_{11}) to solutions containing 10 nM each of annealed S_1(a)-L, R_1(b) and D^j, with j = 5, 6, 7 and 10. After the signal reached a plateau, all reaction wells were treated with large excess of T_{11} to ensure complete consumption of any unreacted monomer in the reaction mixture. The concentrations of the reactants are as given in Table S18.

Table S18. Reaction protocol for catalytic templating with fuels of varying internal toehold lengths for experiments shown in Figure 3a-d in the manuscript.

**Reactants**	**Concentrations in nM**				
S_1-L	10	10	10	10	10
R_1	10	10	10	10	10
D^j	10	10	10	10	10
T_{11} (added by injector)	0	2	3	4	5

**Positive Control**	**Concentrations in nM**			
S_1-R_1-D^7	5	10	15	20

SI.2.5.3 Catalytic templating regulated by excess of fuel molecule

Pre-annealed S_1(a)-L, R_1(b) and 2 nM of T_{11} were reacted with excess of fuel D^7 or D^6 as described in Table S19. To probe the region between 10 nM and 50 nM for D^6 further, additional experiments were performed as described in Table S20. In this experiment, after the signal reached a plateau, all reaction wells were treated with large excess of T_{11} to ensure complete consumption of any unreacted monomer in the reaction mixture.

Table S19. Reaction protocol for control of catalytic turnover with excess concentration of fuel for experiments shown in Figure S9.

**Reactants**	**Concentrations in nM**					
S_1-L	10	10	10	10	10	10
R_1	10	10	10	10	10	10
D^7/D^6	0	5	10	50	100	200
T_{11} (added by injector)	2	2	2	2	2	2

**Positive Controls**	**Concentrations in nM**			
S_1-R_1-D^7	5	10	15	20
S_1-R_1-D^6	5	10	15	20

Table S20. Reaction protocol for control of catalytic turnover with excess concentration of D_6 for experiments shown in Figure 3e in the manuscript.

**Reactants**	**Concentrations in nM**					
S_1-L	10	10	10	10	10	10
R_1	10	10	10	10	10	10
D^6	0	10	20	30	40	50
T_{11} (added by injector)	2	2	2	2	2	2

**Positive Control**	**Concentrations in nM**			
S_1-R_1-D^6	5	10	15	20

SI.2.5.4 Catalytic templating with high monomer-to-template ratio

Pre-annealed S_1(a)-L, R_1(b) and fuel D[7] were reacted with increasing concentrations of the template T_{11}(a). The concentrations of the reactants are given in the table S21.

Table S21. Reaction protocol for experiments demonstrating catalytic turnover with high monomer to template ratio for experiments shown in Figure 3f in the manuscript and S10.

**Reactants**	**Concentrations in nM**				
S_1-L	50	50	50	50	50
R_1	50	50	50	50	50
D	100	100	100	100	100
T_{11} (added by injector)	0	0.5	1.0	1.5	2.0

**Positive Control**	**Concentrations in nM**			
S_1-R_1-D	10	20	50	100

SI.2.5.5 Reactions for orthogonal dimerization

A. Full reaction for orthogonal set 2. Varying concentrations (0-5 nM) of T_{22} were added to 10 nM of each of pre-annealed complex S_2(a)-L, monomer R_2(a) and the fuel D[7]. The concentrations of the reactants are tabulated in table S22.

Table S22. Reaction protocol for the catalytic templating of the orthogonal set 2 for experiments shown in Figure S11.

**Reactants**	**Concentrations in nM**				
S_2-L	10	10	10	10	10
R_2	10	10	10	10	10
D	10	10	10	10	10
T_{22} (added by injector)	0	2	3	4	5

**Positive Control**	**Concentrations in nM**			
S_2-R_2-D	5	10	15	20

B. Dimerization reactions for each pair of monomers triggered with four different templates We first tested specificity by exposing all 4 possible combinations of monomers to each template individually. Pre-annealed S_1(a)-L/S_2(a)-L, R_1(b)/R_2(a) and fuel D were reacted with low concentrations of each template in separate experiments, as detailed in Table S23.

Table S23. Reaction protocol for testing specificity by adding four different templates to each monomer pair for experiments shown in Figure 4a in the manuscript (with S_1-R_1-D) and Figure S12 (with S_1-R_2-D, S_2-R_1-D and S_2-R_2-D).

**Reactants**	**Concentrations in nM**			
S_1-L	10	10	10	10
R_1	10	10	10	10
D	10	10	10	10
Template (added manually)	2 (T_{11})	2 (T_{12})	2 (T_{21})	2 (T_{22})
Matching	*th, hh*	*th*	*hh*	–
Expected nature of reaction	catalytic turnover	only TMSD	no reaction	no reaction

**Positive Control**		**Concentrations in nM**
S_1-R_1-D	5	10

**Reactants**	**Concentrations in nM**			
S_1-L	10	10	10	10
R_2	10	10	10	10
D	10	10	10	10
Template (added manually)	2 (T_{11})	2 (T_{12})	2 (T_{21})	2 (T_{22})
Matching	*th*	*th, hh*	–	*hh*
Expected nature of reaction	only TMSD	catalytic turnover	no reaction	no reaction

**Positive Control**		**Concentrations in nM**
S_1-R_2-D	5	10

Reactants	Concentrations in nM			
S_2-L	10	10	10	10
R_1	10	10	10	10
D	10	10	10	10
Template (added manually)	2 (T_{11})	2 (T_{12})	2 (T_{21})	2 (T_{22})
Matching	*hh*	–	*th, hh*	*th*
Expected nature of reaction	no reaction	no reaction	catalytic turnover	only TMSD

Positive Control		Concentrations in nM
S_2-R_1-D	5	10

Reactants	Concentrations in nM			
S_2-L	10	10	10	10
R_2	10	10	10	10
D	10	10	10	10
Template (added manually)	2 (T_{11})	2 (T_{12})	2 (T_{21})	2 (T_{22})
Matching	–	*hh*	*th*	*th, hh*
Expected nature of reaction	no reaction	no reaction	only TMSD	catalytic turnover

Positive Control		Concentrations in nM
S_2-R_2-D	5	10

C. Specificity of the templates in a mixed pool of monomers. We then tested the specificity of templates by adding each template individually to a mixture of monomers S_1, R_1, S_2 and R_2. The template in question was added into pre-annealed S_1(a)-L, S_2(a)-L; R_1(b), R_2(a) and fuel D. The reaction setup is tabulated in Table S24.

Table S24. Reaction protocol for the test of specificity for each template in a mixture of all four monomers for experiments shown in Figure 4b-c in manuscript.

**Reactants**	**Concentrations in nM**			
S_1-L	10	10	10	10
R_1	10	10	10	10
S_2-L	10	10	10	10
R_2	10	10	10	10
D	10	10	10	10
Template (added manually)	5 (T_{11})	5 (T_{12})	5 (T_{21})	5 (T_{22})

**Positive Controls**	**Concentrations in nM**	
S_1-R_1-D	5	10
S_1-R_2-D	5	10
S_2-R_1-D	5	10
S_2-R_2-D	5	10

D. Gel electrophoresis for specificity of the templates in a mixed pool of monomers. Individual templates were added into pre-annealed S_1(a)-L, S_2(a)-L; R_1(b), R_2(b) and fuel D. The reaction setup is tabulated in Table S25. Products of reactions were run in a PAGE gel alongside pre-annealed controls as described in the methods section of the manuscript.The % yield of the products in the respective lanes with respect to the positive controls are shown in Table S37 in SI.5.5.3.

Table S25. Reaction protocol for the PAGE test of specificity for each template in a mixture of all four monomers for experiments shown in Figures 4e in manuscript and S13. Each column shows the reagents that were added together and then loaded into the gel.

**Reactants**	**Concentrations in nM**									
	Reac^n	Reac^n	Reac^n	Reac^n	Reac^n	Reac^n	Control	Control	Control	Control
S_1-L	10	10	10	10	10	10	–	–	–	–
R_1	10	10	10	10	10	10	–	–	–	–
S_2-L	10	10	10	10	10	10	–	–	–	–
R_2	10	10	10	10	10	10	–	–	–	–
D	–	10	10	10	10	10	–	–	–	–
T_{11}	–	–	2	–	–	–	–	–	–	–
T_{12}	–	–	–	2	–	–	–	–	–	–
T_{21}	–	–	–	–	2	–	–	–	–	–
T_{22}	–	–	–	–	–	2	–	–	–	–
S_1-R_1-D	–	–	–	–	–	–	10	–	–	–
S_1-R_2-D	–	–	–	–	–	–	–	10	–	–
S_2-R_1-D	–	–	–	–	–	–	–	–	10	–
S_2-R_2-D	–	–	–	–	–	–	–	–	–	10

SI.2.5.6 Reactions with upstream gate circuitry

A. Single-input YES gate. The fuel Dn is initially bound to ancillary strand X, in solution with S_1(a)-L and R_1(a). The reaction was triggered with variable concentrations of the template T_{11}(a) and the input I1 was added immediately after injection, as described in Table S26.

Table S26. Reaction protocol for the catalytic templating regulated by a single-input gate system for experiments shown in Figure 6a in manuscript.

**Reactants**	**Concentrations in nM**						
S_1-L	10	10	10	10	10	10	10
R_1	10	10	10	10	10	10	10
Dn-X	10	10	10	10	10	10	10
T_{11} (added by injector)	0	2	3	4	5	5	0
I1 (added manually)	10	10	10	10	10	0	0

**Positive Controls**	**Concentrations in nM**			
S_1-R_1-Dn	5	10	15	20

B. Two-input AND gate Solutions were prepared of S_1(a)-L, R_1(a) and and extended fuel Dn in a complex with ancillary strands E and XE. Also present is I1, except when demonstrating that the absence of I1 leads to no reaction. The reaction was triggered when either 0 nM or 10 nM of I2 and variable concentrations of template T_{11}(a) were injected simultaneously in the reaction mixture. The reaction protocol is tabulated in Table S27.

Table S27. Reaction protocol for the catalytic templating reaction coupled with a 2-input AND gate system for experiments shown in Figure 6b in manuscript.

**Reactants**	**Concentrations in nM**								
S_1-L	10	10	10	10	10	10	10	10	10
R_1	10	10	10	10	10	10	10	10	10
E-Dn-XE	10	10	10	10	10	10	10	10	10
I1	10	10	10	10	10	10	0	0	0
I2 (added by injector)	10	10	10	10	10	0	10	0	0
T_{11} (added by injector)	0	2	3	4	5	5	5	5	0

**Positive Controls**	**Concentrations in nM**			
S_1-R_1-Dn	5	10	15	20

C. Catalytic gate The fuel Dn is initially bound to ancillary strand X, in solution with S_1(a)-L and R_1(a) and Z. The reaction was triggered with a low concentration of $I1_M$ and variable concentrations of the template T_{11}. The reaction protocol is tabulated in Table S28. We also performed additional experiments with high concentrations of the sequestered fuel and monomers, as outlined in in Table S29.

Table S28. Reaction protocol for the catalytic templating coupled with a catalytic gate system for experiments shown in Figure 6c in manuscript.

**Reactants**	**Concentrations in nM**						
S_1-L	10	10	10	10	10	10	10
R_1	10	10	10	10	10	10	10
Dn-X	10	10	10	10	10	10	10
Z	10	10	10	10	10	10	10
$I1_M$ (added by injector)	2	2	2	2	2	0	0
T_{11} (added by injector)	0	2	3	4	5	5	0

**Positive Controls**	**Concentrations in nM**			
S_1-R_1-Dn	5	10	15	20

Table S29. Reaction protocol for the catalytic templating coupled with a catalytic gate, with high monomers to template ratio - for experiments shown in Figure 6e in manuscript.

**Reactants**	**Concentrations in nM**				
S_1-L	50	50	50	50	50
R_1	50	50	50	50	50
Dn-X	100	100	100	100	100
Z	100	100	100	100	100
T_{11} (added by injector)	0	0.5	1.0	1.5	2.0
$I1_M$ (added manually)	2	2	2	2	2

**Positive Controls**	**Concentrations in nM**			
S_1-R_1-Dn	5	10	50	100

D. NOT gate The fuel Dn is initially bound to ancillary strand X, in solution with S_1(a)-L and R_1(a) and Z. The reaction was triggered with a low concentration of $I1_M$, variable concentrations of the template T_{11}, and a large concentration of $I\bar{1}_M$. The precise conditions are tabulated in Table S30. Note that the catalytic experiments without $I\bar{1}_M$ function as a negative control for this experiment.

Table S30. Reaction protocol for catalytic templating coupled with a NOT gate for experiments shown in Figure 6d in manuscript.

**Reactants**	**Concentrations in nM**				
S_1-L	10	10	10	10	10
R_1	10	10	10	10	10
Dn-X	10	10	10	10	10
Z	10	10	10	10	10
$I1_M$ (added by injector)	2	2	2	2	2
T_{11} (added by injector)	0	2	3	4	5
$I\bar{1}_M$ (added manually)	25	25	25	25	25

**Positive Controls**	**Concentrations in nM**			
S_1-R_1-Dn	5	10	15	20

E. Catalytic templating with variable concentrations of input from an upstream gate circuitry The reactions are done in a similar way as that of the single-input YES gate (SI.2.5.6.A) and the catalytic gate (SI.2.5.6.C) except that the input concentrations are varied, as described in Table S31 and S32.

Table S31. Reaction protocol for the catalytic templating regulated by a single-input gate system with variable input as shown in Figure S15a.

**Reactants**	**Concentrations in nM**		
S_1-L	10	10	10
R_1	10	10	10
Dn-X	10	10	10
I1 (added by injector)	0	5	10
T_{11} (added by injector)	2	2	2

**Positive Controls**	**Concentrations in nM**			
S_1-R_1-Dn	5	10	15	20

Table S32. Reaction protocol for the catalytic templating regulated by a catalytic YES gate system with variable input as shown in Figure S15b.

**Reactants**	**Concentrations in nM**		
S_1-L	10	10	10
R_1	10	10	10
Dn-X	10	10	10
Z	10	10	10
$I1_M$ (added by injector)	0	1	2
T_{11} (added by injector)	5	5	5

**Positive Controls**	**Concentrations in nM**			
S_1-R_1-Dn	5	10	15	20

SI.3 Data processing

This section describes how raw fluorescence signals (AFU) are converted into inferred concentrations or into a normalised, dimensionless readout. Section SI.3.2 defines a plate-level normalisation based on positive controls; subsequent subsections apply assay-specific signal models and inference steps. The data processing pipeline is carried out using the in-house software Clearissa v1.71, available at `https://github.com/PrinciplesKJ/Clearissa_1.71`. Step-by-step instructions for reproducing the data processing as described in the following sections are provided in `https://doi.org/10.5281/zenodo.20560387`.

Raw fluorescence signals can exhibit time-dependent changes due to instrument drift and handling steps (for example, sealing, injections, or plate ejection and reinsertion). We assume that the measured fluorescence is the sum of contributions from the individual species present in a reaction well. Each species contribution is proportional to its concentration, and the *ratios* of the corresponding proportionality constants do not change under these perturbations.

SI.3.1 General experimental context and common notation

A “well” refers to an individual reaction well on a plate. Each plate contains the following:

- a buffer-only blank well with raw fluorescence $f_{\text{blank}}(t)$ (AFU),
- an assay-specific negative-control well with raw fluorescence $f_{\text{neg}}(t)$ (AFU),
- one or more positive-control wells containing a fluorescent reference species at known concentration(s) $[C]_q$, with raw fluorescence $f_{\text{pos},q}(t)$ (AFU). Here typically $[C]_q \in \{5, 10, 15, 20\}$ nM, and in some cases $\{10,\ 20,\ 50,\ 100\}$ nM for reactions with high monomer concentrations,
- experimental wells with raw fluorescence $f_{\text{raw}}(t)$ (AFU).

Here, $f_{\text{raw}}(t)$ denotes the raw fluorescence time trace of an arbitrary experimental well; when multiple experimental wells are indexed explicitly we write $f_{\text{raw},i}(t)$.

Unless stated otherwise, background correction is performed by blank subtraction:

$$f_{\text{corr}}(t) = f_{\text{raw}}(t) - f_{\text{blank}}(t), \tag{S1}$$

where $f_{\text{corr}}(t)$ is the blank-subtracted trace (AFU). The same procedure is applied to positive control and negative control traces.

SI.3.1.1 Definitions

- Initialisation window: a 15 min pre-trigger interval

  $$t \in [t_{\text{init,start}},\, t_{\text{init,end}}], \tag{S2}$$

  acquired while wells contain the initial 150 μL mixture (before addition of the remaining 50 μL to reach 200 μL). This window is used to estimate a mini calibration curve.

- Oversaturation window: a 15 min interval after the injection of a saturating level of a strand or complex intended to push one species to react completely

  $$t \in [t_{\text{over,start}},\, t_{\text{over,end}}]. \tag{S3}$$

  It is necessary to wait for a stable (plateau) signal before this window can be taken.

- Main measurement window: the interval used for kinetic analysis, starting at the trigger injection timepoint t_{inj} and ending once the signal has reached a steady state.

  $$t \in [t_{\text{meas,start}},\, t_{\text{meas,end}}], \qquad t_{\text{meas,start}} = t_{\text{inj}}. \tag{S4}$$

Here, $t_{\mathrm{meas,end}}$ is defined as the earliest timepoint at which the trace has reached a stable plateau; for slow reactions, $t_{\mathrm{meas,end}}$ is set to the final recorded timepoint.

- $\mathrm{pos}_{q,\mathrm{init}}$: mean blank-subtracted positive-control fluorescence in the initialisation window (AFU). The index q refers to the respective known concentration of the positive control.
- c_{io}: fluorescence-to-concentration slope (AFU per nM) of the positive control, determined from the initialisation window.
- C_{ref}: reference concentration used when a concentration-referenced trajectory is required (typically 10 nM).
- $r_q(t)$: signal drift ratio for positive control q (dimensionless).

SI.3.2 Positive control normalisation

Positive control normalisation defines a time-dependent, background-corrected reference trajectory $p_{\mathrm{corr}}(t)$ (AFU) that corrects plate-wide, time-dependent artifacts (gain drift, handling steps, plate ejection/reinsertion).

SI.3.2.1 Procedure

A. Blank subtraction. We first correct the positive control for the background blank signal.

$$f_{\mathrm{pos},q,\mathrm{corr}}(t) = f_{\mathrm{pos},q}(t) - f_{\mathrm{blank}}(t). \tag{S5}$$

B. Calibration slope from the initialisation window. A mini calibration of fluorescence against concentration for the positive control is formed by linear regression of $\mathrm{pos}_{q,\mathrm{init}}$ against $[C]_q$:

$$\mathrm{pos}_{q,\mathrm{init}} = c_{\mathrm{io}}\,[C]_q. \tag{S6}$$

We thus assume a no-intercept model after blank subtraction. For the purposes of our analysis, c_{io} is taken as the ground truth with which other concentrations can be converted to and from fluorescence.

C. Plate-level drift profile. Each positive-control trace is converted to a ratio relative to its own initialisation mean:

$$r_q(t) = \frac{\mathrm{pos}_{q,\mathrm{corr}}(t)}{\mathrm{pos}_{q,\mathrm{init}}}. \tag{S7}$$

The signal drift profile is the mean across all Q positive controls:

$$r_{\mathrm{mean}}(t) = \frac{1}{Q}\sum_{q=1}^{Q} r_q(t). \tag{S8}$$

D. Reference trajectory in AFU. The information from all positive controls can be used to define a time-dependent nominal positive control fluorescence signal at any concentration C_{ref} according to:

$$p_{\mathrm{corr}}(t) = c_{\mathrm{io}}\, C_{\mathrm{ref}}\, r_{\mathrm{mean}}(t). \tag{S9}$$

Equivalently,

$$p_{\mathrm{corr}}(t) = c_{\mathrm{io}}\, C_{\mathrm{ref}}\, \frac{1}{Q}\sum_{q=1}^{Q} \frac{f_{\mathrm{pos},q}(t) - f_{\mathrm{blank}}(t)}{f_{\mathrm{pos},q,\mathrm{init}}}. \tag{S10}$$

E. Applying the normalisation. For an experimental well with raw fluorescence trace $f_{\mathrm{raw}}(t)$ (AFU), we first apply blank subtraction (Eq. S1) and then divide by $p_{\mathrm{corr}}(t)$ to give a ratio of the signal to the positive control:

$$g(t) = \frac{f_{\mathrm{corr}}(t)}{p_{\mathrm{corr}}(t)}. \tag{S11}$$

When concentration-referenced units are required, we define $h(t)$ with units of nM:

$$h(t) = g(t)\, C_{\mathrm{ref}}. \tag{S12}$$

$h(t)$ gives the concentration of positive control species that would be required to explain the observed fluorescence in the experimental trace.

SI.3.3 Conversion of AFU to concentrations for TMSD reactions (step 1)

SI.3.3.1 Experimental context and signal model

The assay follows step 1, a toehold-mediated strand displacement reaction of the form:

$$\mathrm{S_1\text{-}L} + \mathrm{T_{11}} \rightarrow \mathrm{S_1\text{-}T_{11}} + \mathrm{L}. \tag{S13}$$

Reaction wells were initially loaded with a fluorophore-quencher duplex S_1-L. The non-labelled invader strand T_{11} was injected to initiate the reaction in which L is displaced and S_1-T_{11} is formed. S_1-L exhibits residual fluorescence due to incomplete quenching, whereas the displacement product S_1-T_{11} is fully fluorescent.

Positive-control wells contain S_1-T_{11} at known concentrations, which are used to construct $p_{\mathrm{corr}}(t)$.

We apply the common preprocessing described in SI.3.2 to obtain $h(t)$ (nM) for each experimental trace (Eq. S12), i.e.

$$h(t) = \frac{f_{\mathrm{raw}}(t) - f_{\mathrm{blank}}(t)}{p_{\mathrm{corr}}(t)}\, C_{\mathrm{ref}}. \tag{S14}$$

For concentration inference we assume

$$h(t) = [\mathrm{S_1\text{-}T_{11}}] + \beta\, [\mathrm{S_1\text{-}L}], \tag{S15}$$

where β is the fractional fluorescence of S_1-L relative to S_1-T_{11} (dimensionless). We further

assume mass conservation

$$[\mathrm{S_1\text{-}L}]_0 = [\mathrm{S_1\text{-}L}] + [\mathrm{S_1\text{-}T_{11}}], \tag{S16}$$

so that

$$h(t) = [\mathrm{S_1\text{-}T_{11}}](1-\beta) + \beta\,[\mathrm{S_1\text{-}L}]_0, \tag{S17}$$

and thus

$$[\mathrm{S_1\text{-}T_{11}}] = \frac{h(t) - \beta[\mathrm{S_1\text{-}L}]_0}{1-\beta}. \tag{S18}$$

SI.3.3.2 Concentration inference procedure (per trace)

A. Determine the initial substrate concentration $[\mathrm{S_1\text{-}L}]_0$ by oversaturation. Under oversaturation by injection of excess $\mathrm{T_{11}}$ we assume full conversion of $\mathrm{S_1}$-L to $\mathrm{S_1\text{-}T_{11}}$, hence $h(t) = [\mathrm{S_1\text{-}L}]_0$ in the oversaturated plateau window. We estimate

$$[\mathrm{S_1\text{-}L}]_0 = \mathrm{mean}(h(t)) \quad \text{for } t \in [t_{\mathrm{over,start}},\, t_{\mathrm{over,end}}]. \tag{S19}$$

B. Estimate β from the pre-trigger window. In the initialisation window, $[\mathrm{S_1\text{-}T_{11}}] = 0$ and $h(t) = \beta[\mathrm{S_1\text{-}L}]_0$. We compute

$$h_{\mathrm{init}} = \mathrm{mean}(h(t)) \quad \text{for } t \in [t_{\mathrm{init,start}},\, t_{\mathrm{init,end}}], \tag{S20}$$

and estimate

$$\beta = \frac{h_{\mathrm{init}}}{[\mathrm{S_1\text{-}L}]_0}. \tag{S21}$$

This estimate of β is calculated separately for each reaction well.

C. Reconstruct concentrations over time Having identified the constants $[S_1\text{-}L]_0$ and β, Eq. S18 can be used to infer the concentrations of the product $[\mathrm{S_1\text{-}T_{11}}]$ over time.

SI.3.4 Conversion of AFU to concentrations for HMSD reactions (step 2)

SI.3.4.1 Experimental context and signal model

HMSD (step 2) was monitored in the donor channel of a donor-acceptor FRET pair. The donor-labelled complex S_1-T_{11} was initially present in solution and provides the bright donor reference signal. Upon a HMSD reaction with R_1, a ternary complex forms in which donor emission was quenched by FRET:

$$S_1\text{-}T_{11} + R_1 \rightarrow S_1\text{-}T_{11}\text{-}R_1. \tag{S22}$$

Consequently, the donor-channel fluorescence decreases as product accumulates.

We initially considered inferring product formation from the FRET readout; however, in practice the donor-channel signal provided a stronger signal-to-noise ratio and is therefore used for concentration inference.

A donor reference control containing pre-annealed S_1-T_{11} at a known concentration of 10 nM was used for donor-channel normalisation. Note that this control contains 0 nM product and is therefore not the positive control in the sense it is in the TMSD conversion model. The donor reference control serves as the normalisation reference rather than as a baseline to be subtracted, and is therefore not plotted alongside the experimental product traces.

Because only a single S_1-T_{11} reference trace is available, $h(t)$ is obtained by blank subtraction followed by pointwise normalisation to the blank-subtracted S_1-T_{11} reference trajectory and scaling by its known concentration.

We apply blank subtraction in the donor channel to obtain

$$f_{\text{corr}}(t) = f_{\text{raw}}(t) - f_{\text{blank}}(t), \qquad f_{\text{ref,corr}}(t) = f_{\text{ref}}(t) - f_{\text{blank}}(t), \tag{S23}$$

where $f_{\mathrm{raw}}(t)$ is the experimental donor-channel trace and $f_{\mathrm{ref}}(t)$ is the donor reference-control trace (pre-annealed $\mathrm{S_1}$-$\mathrm{T_{11}}$). In the HMSD workflow, $f_{\mathrm{ref,corr}}(t)$ serves the same functional role as $p_{\mathrm{corr}}(t)$ in the TMSD conversion, but is taken directly from a single donor reference control rather than being constructed from multiple positive-control wells. We then form the pointwise ratio

$$g(t) = \frac{f_{\mathrm{corr}}(t)}{f_{\mathrm{ref,corr}}(t)}, \tag{S24}$$

and define the concentration-referenced donor signal

$$h(t) = g(t)\,[\mathrm{S_1}\text{-}\mathrm{T_{11}}]_{\mathrm{ref}}, \tag{S25}$$

where $[\mathrm{S_1}\text{-}\mathrm{T_{11}}]_{\mathrm{ref}}$ is the known concentration of the donor reference control.

Concentration inference uses the linear signal model

$$h(t) = [\mathrm{S_1}\text{-}\mathrm{T_{11}}](t) + \beta\,[\mathrm{S_1}\text{-}\mathrm{T_{11}}\text{-}\mathrm{R_1}](t), \tag{S26}$$

where β is the residual donor brightness of the product $\mathrm{S_1}$-$\mathrm{T_{11}}$-$\mathrm{R_1}$ relative to $\mathrm{S_1}$-$\mathrm{T_{11}}$. Assuming conservation of the donor-labelled complex,

$$[\mathrm{S_1}\text{-}\mathrm{T_{11}}]_0 = [\mathrm{S_1}\text{-}\mathrm{T_{11}}](t) + [\mathrm{S_1}\text{-}\mathrm{T_{11}}\text{-}\mathrm{R_1}](t), \tag{S27}$$

we obtain

$$[\mathrm{S_1}\text{-}\mathrm{T_{11}}\text{-}\mathrm{R_1}](t) = \frac{[\mathrm{S_1}\text{-}\mathrm{T_{11}}]_0 - h(t)}{1-\beta}. \tag{S28}$$

SI.3.4.2 Concentration inference procedure

Compared to the TMSD concentration inference procedure (Sec. SI.3.3), HMSD differs in three respects: (i) product formation decreases the donor-channel fluorescence due to quenching; (ii) the donor reference control is a single-concentration $\mathrm{S_1}$-$\mathrm{T_{11}}$ trace used for pointwise

normalisation; and (iii) the quenching factor β is estimated from separate pre-annealed S_1-T_{11} and S_1-T_{11}-R_1 control wells.

A. Estimate β from control wells. The residual brightness factor β is estimated from donor-channel measurements of pre-annealed control complexes. The unquenched donor reference control S_1-T_{11} is typically measured in a single well at a known concentration (typically $[S_1\text{-}T_{11}]_{\text{ref}} = 10$ nM); after blank subtraction its donor-channel signal is denoted $f_{\text{ref,corr}}(t)$. The quenched product control S_1-T_{11}-R_1 is typically measured in four wells at different concentrations and is processed using the positive control normalisation procedure (Sec. SI.3.2) to yield a drift-corrected reference trajectory in the donor channel, denoted $p_{\text{corr,prod}}(t)$.

We compute β from the ratio of initialisation-window means,

$$\beta = \frac{\text{mean}(p_{\text{corr,prod}}(t))}{\text{mean}(f_{\text{ref,corr}}(t))} \quad \text{for } t \in [t_{\text{init,start}},\, t_{\text{init,end}}]. \tag{S29}$$

Because both $p_{\text{corr,prod}}(t)$ and $f_{\text{ref,corr}}(t)$ are expressed on the same concentration-referenced scale (using the same reference concentration, typically 10 nM), the choice of reference concentration cancels in the ratio and β is treated as concentration-independent. For a given plate (experiment), the resulting β is treated as a fixed constant and applied to all experimental wells on that plate.

B. Estimate $[S_1\text{-}T_{11}]_0$ and reconstruct product concentrations. Prior to reaction initiation, no product has formed, so $[S_1\text{-}T_{11}\text{-}R_1](t) = 0$ and therefore $h(t) = [S_1\text{-}T_{11}]_0$ in the initialisation window. We estimate

$$[S_1\text{-}T_{11}]_0 = \text{mean}(h(t)) \quad \text{for } t \in [t_{\text{init,start}},\, t_{\text{init,end}}], \tag{S30}$$

and then use Eq. S28 to infer $[S_1\text{-}T_{11}\text{-}R_1](t)$. Note that $[S_1\text{-}T_{11}]_0$ is calculated individually for every trace. Due to measurement noise, inferred product concentrations slightly below zero (down to -1 nM) are permitted.

SI.3.5 Conversion of AFU to concentrations for internal TMSD reactions (step 3)

SI.3.5.1 Experimental context and signal model

Internal TMSD follows:

$$D + S_1\text{-}T_{11}\text{-}R_1 \rightarrow S_1\text{-}R_1\text{-}D + T_{11}. \tag{S31}$$

Fuel D was pre-incubated in the wells before the reaction starts, and $S_1\text{-}T_{11}\text{-}R_1$ is injected at time t_{inj}. The injection produces an instantaneous fluorescence jump due to residual $S_1\text{-}T_{11}\text{-}R_1$ fluorescence (incomplete quenching). This injection offset is corrected using a negative control well containing $S_1\text{-}T_{11}\text{-}R_1$ only (no fuel).

Positive-control wells contain the fully fluorescent product species $S_1\text{-}R_1\text{-}D$ at known concentrations, and these traces are used to construct $p_{\text{corr}}(t)$ for the channel analysed here (Sec. SI.3.2).

We apply the same procedure as in SI.3.3, namely assuming

$$[S_1\text{-}R_1\text{-}D] = \frac{h(t) - \beta[S_1\text{-}T_{11}\text{-}R_1]_0}{1-\beta}, \tag{S32}$$

and estimating the constants β and $[S_1\text{-}T_{11}\text{-}R_1]_0$. $[S_1\text{-}T_{11}\text{-}R_1]_0$ can be inferred from the signal after oversaturation with excess D, which drives complete conversion of $S_1\text{-}T_{11}\text{-}R_1$ to $S_1\text{-}R_1\text{-}D$ in each experimental well.

$$[S_1\text{-}T_{11}\text{-}R_1]_0 = \text{mean}(h_{\text{exp}}(t)) \quad \text{for } t \in [t_{\text{over,start}},\, t_{\text{over,end}}]. \tag{S33}$$

However, the initial time points cannot be used to estimate β because the S_1-T_{11}-R_1 complex was not in solution prior to injection. We therefore use a negative control well (containing S_1-T_{11}-R_1 but no D), in which no product forms and thus $h_{\text{neg}}(t) = \beta[S_1\text{-}T_{11}\text{-}R_1]_0$. We define

$$h_{\text{neg,base}} = \text{mean}(h_{\text{neg}}(t)) \quad \text{for } t \in [t_{\text{nc,start}},\, t_{\text{nc,end}}], \tag{S34}$$

where $[t_{\text{nc,start}},\, t_{\text{nc,end}}]$ refers to a 30 min interval selected after signal stabilisation in the negative-control trace.

To obtain a single β for the experiment, we use a robust plate-level estimate of the initial substrate concentration, $[S_1\text{-}T_{11}\text{-}R_1]_{0,\text{plate}}$, defined as the median of $[S_1\text{-}T_{11}\text{-}R_1]_{0,\text{well}}$ across experimental wells. A single value of β is then estimated as

$$\beta = \frac{h_{\text{neg,base}}}{[S_1\text{-}T_{11}\text{-}R_1]_{0,\text{plate}}}, \tag{S35}$$

and this value is applied to all experimental wells for the experiment. This differs from the standard TMSD analysis, where β is estimated separately for each reaction well.

Having identified the constants $[S_1\text{-}T_{11}\text{-}R_1]_0$ per well and a global β, Eq. S32 can be used to infer the concentrations of the product $[S_1\text{-}R_1\text{-}D]$ over time.

SI.3.6 Calculation of a control-normalised readout in relative units

SI.3.6.1 Experimental context

This mode reports a dimensionless normalised readout (relative fluorescence, RF) rather than concentrations. It is used when a fluorescence-to-concentration mapping is not justified (for example, complex stoichiometry, multiple fluorescent species, or unknown quenching fractions). The essential principle is to report the fluorescence on a scale normalised with respect to two controls – typically a negative control assigned the value 0 and a positive

control assigned the value 1. Note that the system being reported need not contain only the fluorescent species that define these controls.

For each well, we consider the blank-corrected fluorescence $f_{\mathrm{corr}}(t)$ (Eq. S1) and the averaged, blank-corrected positive control $p_{\mathrm{corr}}(t)$ (Eq. S9). We also consider a blank-corrected negative control

$$f_{\mathrm{neg,corr}}(t) = f_{\mathrm{neg}}(t) - f_{\mathrm{blank}}(t). \tag{S36}$$

All quantities are defined for the time period corresponding to the main measurement interval

$$t \in [t_{\mathrm{meas,start}},\ t_{\mathrm{meas,end}}]. \tag{S37}$$

A. Negative-control baseline (AFU). A (typically 30 min) baseline window $t \in [t_{\mathrm{nc,start}}, , t_{\mathrm{nc,end}}]$ within the main measurement interval $t \in [t_{\mathrm{meas,start}}, , t_{\mathrm{meas,end}}]$ is selected after any initial transients in the negative control due to the reaction initiation procedure have subsided. We use this time period to define a fixed negative control baseline

$$f_{\mathrm{neg,base}} = \mathrm{mean}(f_{\mathrm{neg,corr}}(t)) \quad \text{for } t \in [t_{\mathrm{nc,start}},\ t_{\mathrm{nc,end}}]. \tag{S38}$$

B. Relative fluorescence calculation. We then calculate a relative fluorescence as

$$\mathrm{RF}(t) = \frac{f_{\mathrm{corr}}(t) - f_{\mathrm{neg,base}}}{p_{\mathrm{corr}}(t) - f_{\mathrm{neg,base}}}. \tag{S39}$$

Note that this approach ignores (typically very small) changes in the negative control signal over the main measurement window $t \in [t_{\mathrm{meas,start}},\ t_{\mathrm{meas,end}}]$.

SI.4 Kinetic model fitting

This section describes how the inferred concentration traces from our experiments are fitted to kinetic models. The data fitting pipeline is performed using in-house software Clearissa v1.71, available at `https://github.com/PrinciplesKJ/Clearissa_1.71`. The specific step by step instructions to reproduce the fits are outlined in `https://doi.org/10.5281/zenodo.20560387`.

SI.4.1 Single step bimolecular reactions

This section describes how concentration trajectories for TMSD, internal toehold TMSD and HMSD,inferred from fluorescence (Secs. SI.3.3, SI.3.5 and SI.3.4) are fitted to a standard mass-action model. Fits are performed only over a user-defined kinetic window $t \in [t_0, t_{\text{meas,end}}]$, where t_0 is the reaction-onset time chosen in the analysis software, typically aligned to the trigger injection and excluding any pre-onset baseline. The model assumes well-mixed mass-action kinetics and constant volume over the fitted window.

All single-step reactions are modelled as an irreversible bimolecular conversion of the form

$$\mathrm{X} + \mathrm{Z} \to \mathrm{Y} + \mathrm{W}, \tag{S40}$$

where Y is the product species concentration trajectory to be fitted. W is a placeholder for any byproduct(s) that are created within the reaction but are not included in the fitted rate law. The fitted observable is $y(t) = [\mathrm{Y}](t)$.

The ordinary differential equation governing all three reactions is expected to be of the form

$$\frac{d}{dt}[\mathrm{Y}](t) = +k_f[\mathrm{X}](t)[\mathrm{Z}](t), \tag{S41}$$

where Y is the product and X and Z are the reactants. The conservation of mass

$$[\mathrm{X}](t) = [\mathrm{X}]_0 - [\mathrm{Y}](t), \tag{S42}$$

$$[\mathrm{Z}](t) = [\mathrm{Z}]_0 - [\mathrm{Y}](t), \tag{S43}$$

also applies. Substituting these conservation laws yields the reduced one-dimensional form used for fitting,

$$\frac{d}{dt}[\mathrm{Y}](t) = k_f\big([\mathrm{X}]_0 - [\mathrm{Y}](t)\big)\big([\mathrm{Z}]_0 - [\mathrm{Y}](t)\big). \tag{S44}$$

For each experimental trace, we fit a modelled trajectory $y_{\mathrm{sim}}(t)$ to the experimentally inferred $[\mathrm{Y}](t)$, using k_f and $[\mathrm{X}]_0$ as fitting parameters and using the value of $[\mathrm{Z}]_0$ calculated when inferring $[\mathrm{Y}](t)$.

SI.4.1.1 Mapping to assay-specific species.

Below, we state which species corresponds to X, Y, Z in each experiment that is fitted.

A. Standard TMSD (Sec. SI.3.3).

$$\mathrm{X} \equiv \mathrm{T}_{11}, \qquad \mathrm{Z} \equiv \mathrm{S}_1\text{-L}, \qquad \mathrm{Y} \equiv \mathrm{S}_1\text{-}\mathrm{T}_{11}, \qquad \mathrm{W} \equiv \mathrm{L}, \qquad y(t) = [\mathrm{S}_1\text{-}\mathrm{T}_{11}](t). \tag{S45}$$

B. HMSD (Sec. SI.3.4).

$$\mathrm{X} \equiv \mathrm{R}_1, \qquad \mathrm{Z} \equiv \mathrm{S}_1\text{-}\mathrm{T}_{11}, \qquad \mathrm{Y} \equiv \mathrm{S}_1\text{-}\mathrm{T}_{11}\text{-}\mathrm{R}_1, \qquad \mathrm{W} \equiv \emptyset, \qquad y(t) = [\mathrm{S}_1\text{-}\mathrm{T}_{11}\text{-}\mathrm{R}_1](t). \tag{S46}$$

C. Internal TMSD (Sec. SI.3.5).

$$X \equiv D, \quad Z \equiv S_1\text{-}T_{11}\text{-}R_1, \quad Y \equiv S_1\text{-}R_1\text{-}D, \quad W \equiv T_{11}, \quad y(t) = [S_1\text{-}R_1\text{-}D](t). \qquad (S47)$$

SI.4.1.2 Fitting procedure

Fits are performed over the fitting window $t \in [t_0, t_{\text{meas,end}}]$, where $t_0 > t_{\text{meas,start}} = t_{\text{inj}}$ is the reaction-onset time chosen in the analysis software (typically aligned to the trigger injection and excluding any pre-onset baseline). For a candidate parameter set k_f, $[X]_0$ and fixed $[Z]_0$, the model prediction is evaluated using the analytical solution of the reduced mass-action model. Parameter estimation is performed by nonlinear least squares in Python using `scipy.optimize.curve_fit` with non-negativity bounds, fitting $y_{\text{sim}}(t)$ to the inferred product trace $[Y](t)$ over the fitted time points. The default initial guess for the rate constant is $k_f = 10^5$ $M^{-1}s^{-1}$, and the initial guess for $[X]_0$ is 10 nM. Fit quality is quantified using the coefficient of determination R^2, computed from the residual sum of squares between $[Y](t)$ and $y_{\text{sim}}(t)$, normalised by the total sum of squares of $[Y](t)$ about its mean over the fitted time points.

SI.4.1.3 Inference of a representative rate constant from replicate fits.

Replicate traces sharing the same nominal initial conditions are grouped together. Each trace in a group is fitted individually, yielding a rate constant $k_{f,i}$ and fitted concentration $X_{0,i}$ for trace i. The fixed concentration $[Z]_{0,i}$ (obtained during concentration inference) may also vary slightly across replicates due to experimental variations.

For each group, the representative rate constant is taken as the arithmetic mean of the replicate values $k_{f,i}$. Representative concentrations are defined analogously as the arithmetic means of $[X]_{0,i}$ and $[Z]_{0,i}$ across replicates. For visualisation, we plot the group mean experimental trajectory and a shaded envelope spanning the pointwise minimum and maximum

across replicate traces. The group fit trajectory is obtained by simulating the kinetic model using the averaged parameters.

A representative rate constant for each of the reactions as a whole, along with its associated error, which is the standard error of the mean (SEM), are reported in Tables S33 - S35.

SI.4.2 Kinetic model fitting of catalytic turnover experiments

We fit the normalized fluorescence in Figures 3a-d in the manuscript using a very rough model that assumes: (a) that the normalized fluorescence is proportional the concentration of product (we do not distinguish between template-bound and free in solution here); and (b) the template reaches a quasi-steady state in which it operates in a Michaelis-Menten-like way, with a turnover per template given by

$$\frac{d}{dt}[\mathrm{S_1\text{-}R_1\text{-}D}](t) = \frac{k\,[\mathrm{S_1\text{-}L}]^n}{K + [\mathrm{S_1\text{-}L}]^n}\,[T], \tag{S48}$$

where n, k and K are constants and $[T]$ is the template concentration. Here, $[\mathrm{S_1\text{-}L}]$ is being used as a stand-in for any/all the components of the product, since $[\mathrm{S_1\text{-}L}] \approx [\mathrm{S}] \approx [\mathrm{D}]$ in this particular setup.

We expect that the reaction proceeds in a step-by-step fashion, with first S_1 binding to T_{11}, then to R_1, and finally to D forming the completed structure. Each of these steps should be linear in the concentration of the reactants, and therefore we assume that $n = 1$ henceforth, even though the product has three components.

When the templates were injected into the solution, there is a burst of S_1 binding to T_{11} and becoming fluorescent, before the system settles down to a state in which subsequent fluorescence increases require full catalytic turnover. This initial burst is not captured by the model in Eq. S48. Therefore, fits are performed only after a manually chosen start timepoint t_0 at which the trace transitions from the burst regime into a slower regime in

which growth is driven by turnover, consistent with the model in Eq. S48. Within a given experiment, the same t_0 is applied to all traces (common reaction onset), while t_0 may differ between experiments.

SI.4.2.1 Fitting procedure

For each trace, we take the relative fluorescence RF(t) and convert it to an estimated concentration $[\mathrm{S_1\text{-}R_1\text{-}D}](t)$, by multiplying by the (assumed known) reference concentration

$$[\mathrm{S_1\text{-}R_1\text{-}D}](t) = \mathrm{RF}(t) C_{\mathrm{ref}}. \tag{S49}$$

Template concentrations $[T]$ are assumed to match the intended value $(0, 2, 3, 4, 5\ \mathrm{nM})$ for each trace. The fitting window is $[t_0, t_{\mathrm{meas,end}}]$, where t_0 is chosen by inspection. The initial condition is set from the measured value at the first time point within the fitting window

$$y_{\mathrm{sim}}(t_0) = [\mathrm{S_1\text{-}R_1\text{-}D}](t_0), \tag{S50}$$

and is not fitted.

For each system (internal toehold length), we simultaneously fit all traces (including replicates) at all concentrations of template. We use a single k and K parameter shared for all traces, but each trace has its own fitted initial pool of building blocks $[\mathrm{S_1\text{-}L}]_0$. We assume mass conservation for the building-block pool, so that the available pool at time t is $[\mathrm{S_1\text{-}L}]_0 - y_{\mathrm{sim}}(t)$.

For a candidate parameter set, the ODE

$$\frac{d}{dt} y_{\mathrm{sim}}(t) = \frac{k\left([\mathrm{S_1\text{-}L}]_0 - y_{\mathrm{sim}}(t)\right)}{K + \left([\mathrm{S_1\text{-}L}]_0 - y_{\mathrm{sim}}(t)\right)} [T] \tag{S51}$$

with initial condition $y_{\mathrm{sim}}(t_0) = [\mathrm{S_1\text{-}R_1\text{-}D}](t_0)$ is numerically integrated for each trace over $[t_0, t_{\mathrm{meas,end}}]$. Numerical integration is performed in Python using *scipy.integrate.odeint* (LSODA).

Parameter estimation is performed by nonlinear least squares in Python using `scipy.optimize.least_squares` by minimising the residuals between $y_{\mathrm{sim},i}(t)$ and $[\mathrm{S_1\text{-}R_1\text{-}D}](t)$ over the fitted time points. Residuals are computed per trace on its fitted time grid and concatenated across traces for simultaneous optimisation. All fitted parameters are constrained to be non-negative.

Initial guesses are $k = 1$ min^{-1}, $K = 10$ nM, and $[\mathrm{S_1\text{-}L}]_0 = 10$ nM for each trace. The boundaries set for fitting are $k \in [10^{-10}, 1]$ min^{-1}, $K \in [0.5, 500]$ nM, and $[\mathrm{S_1\text{-}L}]_0$ within ±20% of the initial guess. These bounds were chosen to span plausible ranges given the experimental conditions.

For each system, we simultaneously fit all traces (including replicates) at all template concentrations. All traces within a fit share a common fitting window $[t_0, t_{\mathrm{meas,end}}]$, a single k and K, while each trace has its own fitted $[\mathrm{S_1\text{-}L}]_0$.

Fit quality is quantified using the standard sum of squared residuals (least-squares error) over the fitted time points, and the corresponding fitted trajectories are inspected against the experimental traces.

The kinetic fits for the variants of $D^{\mathrm{j}}, j = 5, 6, 10$ indicate that only the ratio k/K is well constrained, consistent with a reaction that operates in a non-saturating regime in which $s(t) = ([\mathrm{S_1\text{-}L}]_0 - y_{\mathrm{sim}}(t)) \ll K$ over the fitted window. For these systems. we therefore reduced Eq. S51 to a linear form to obtain

$$\frac{d}{dt} y_{\mathrm{sim}}(t) = k_{\mathrm{eff}} \left([\mathrm{S_1\text{-}L}]_0 - y_{\mathrm{sim}}(t) \right) [T], \qquad k_{\mathrm{eff}} \equiv \frac{k}{K}, \tag{S52}$$

which removes the k–K degeneracy. For these variants only, we therefore report fits in terms

of the effective rate constant k_{eff}.

SI.5 Supplementary results

SI.5.1 Individual steps

SI.5.1.1 Step 1 – toehold-mediated strand displacement

Data for TMSD using a 6 nt toehold is shown in Figure S2a, alongside data corresponding to a 7 nt toehold in Figure S2b. Both datasets are obtained from single replicas.

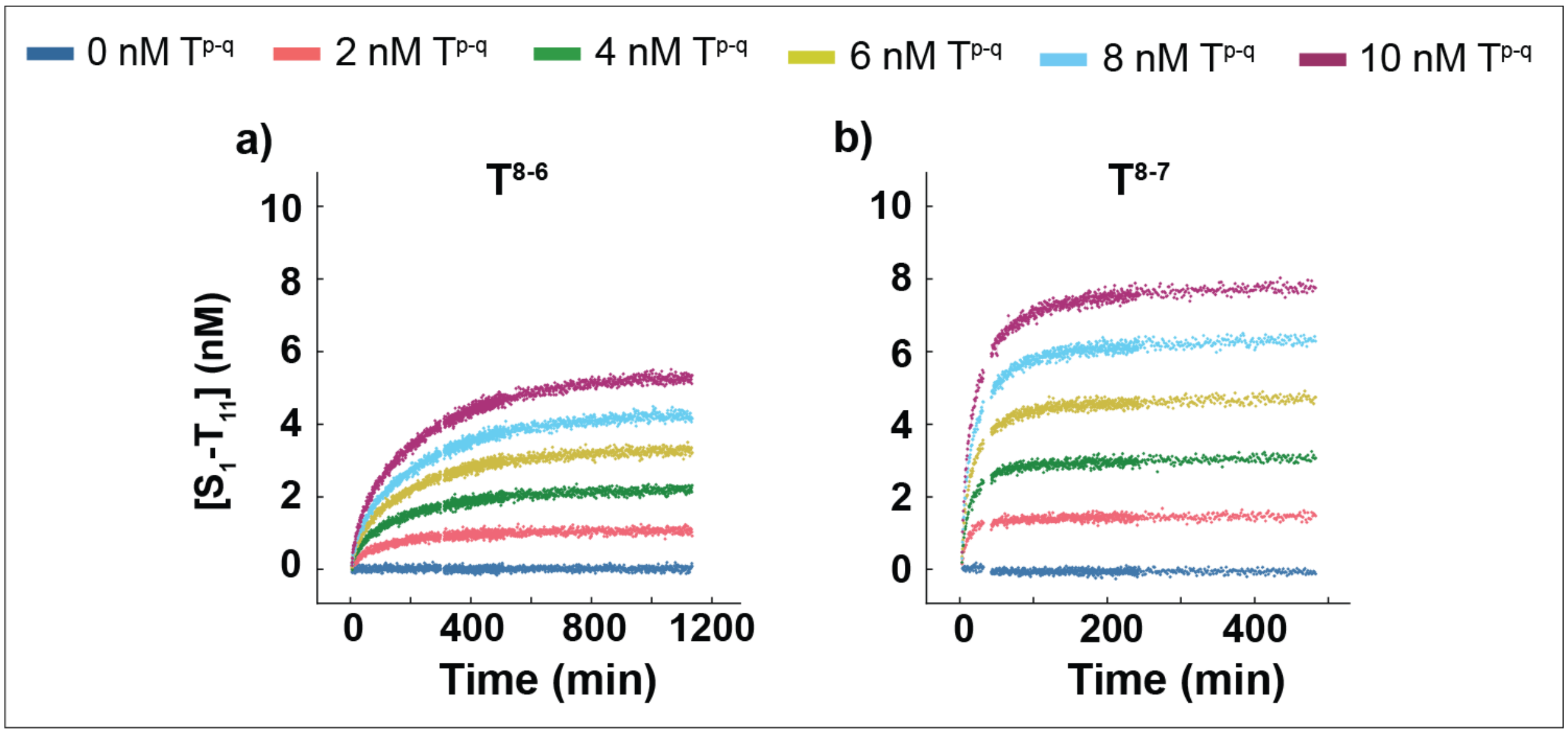

Figure S2. Reaction progress against time for TMSD with varying lengths of toehold. Reactions were conducted according to the experimental protocol outlined in Table S12, with $T^{p\text{-}q}$ added to a solution of S_1-L. Fluorescence in the ATTO 488 channel is converted into product concentration as described in section SI.3.3.

SI.5.1.2 Step 2 – handhold-mediated strand displacement

Data for HMSD using a 0 and 7-9 nt handhold is shown in Figure S3. All datasets are obtained from single replicas.

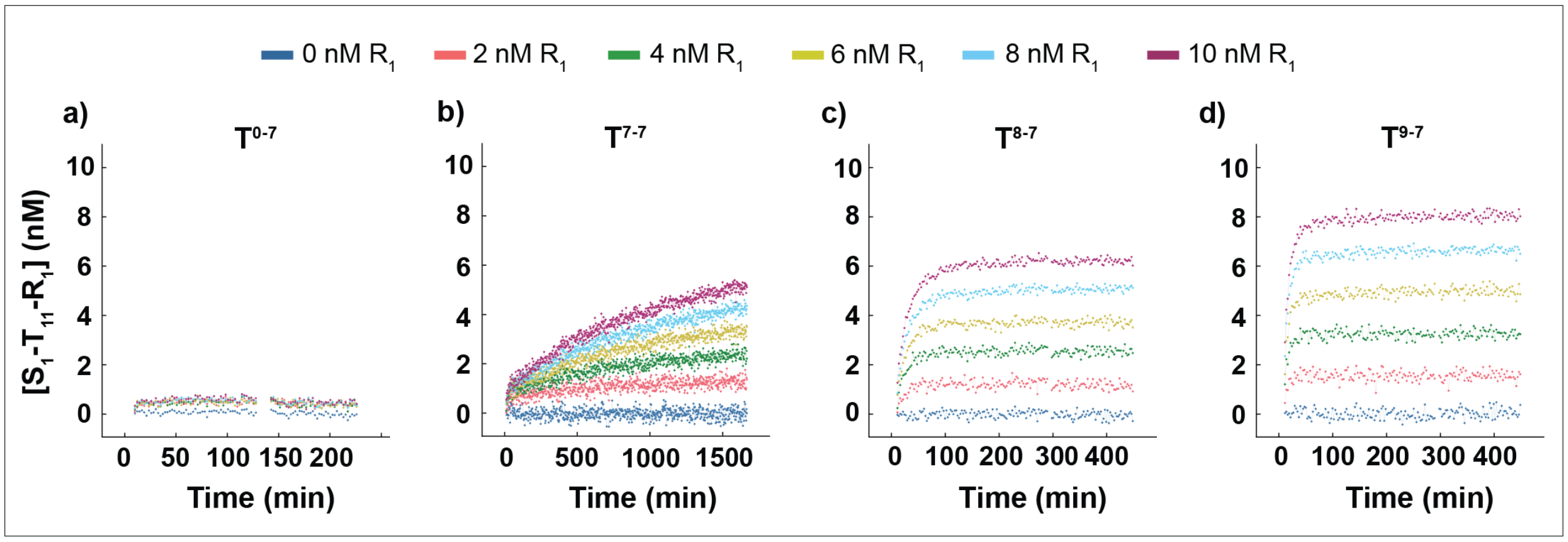

Figure S3. Reaction progress against time for HMSD with varying lengths of handhold. Reactions were conducted according to the experimental protocol outlined in Table S13, with R_1 added to a solution of S_1-$T^{p\text{-}q}$. Fluorescence in the donor channel ATTO 488 is converted into product concentration as described in section SI.3.4.

SI.5.1.3 Step 3 – internal toehold-mediated strand displacement

In addition to the data in Figure 2c in the manuscript, data for internal TMSD using 5, 6, 7 and 10 nt internal toehold are shown in Figure S4a, b, c and d respectively. All datasets are obtained from three replicas with the mean and range plotted in each case.

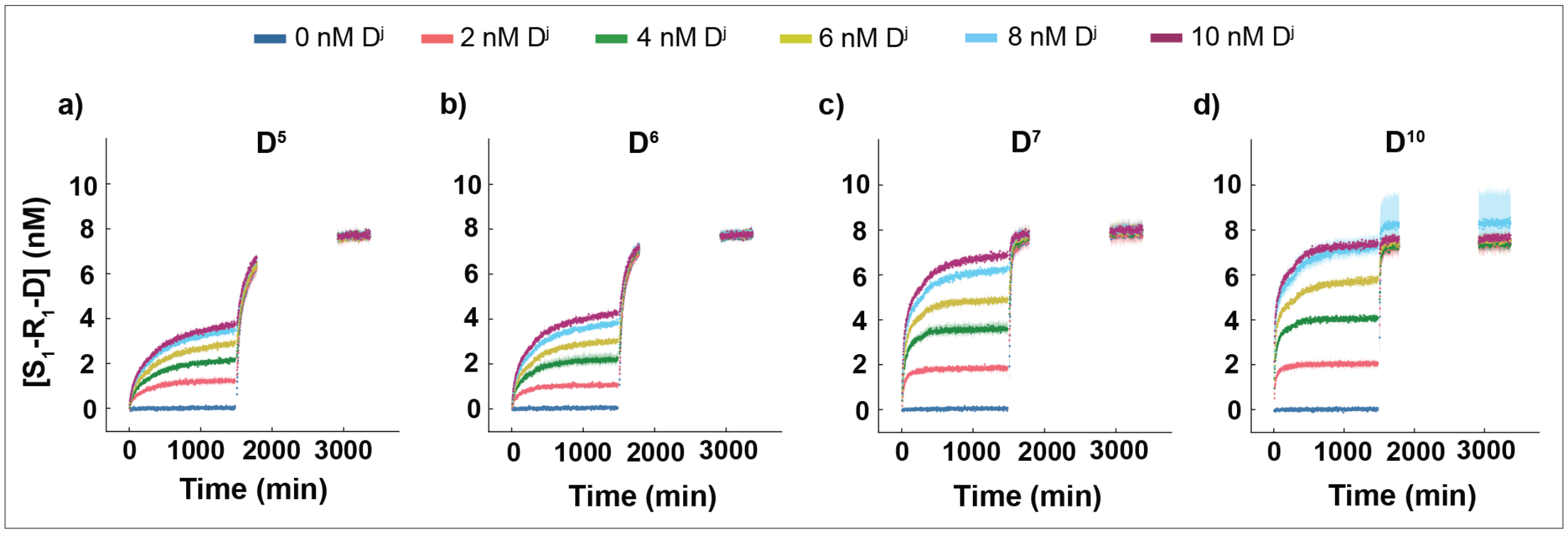

Figure S4. Reaction progress against time for internal TMSD with varying lengths of internal toehold. Reactions were conducted according to the experimental protocol outlined in Table S14, with S_1-R_1-T_{11} added to a solution of D^j. Fluorescence in ATTO 488 channel is converted into product concentration as described in section SI.3.5.

Table S33. Observed second-order rate constants (k_f) for each trace of the 3 sets of replicas (A01-A06, A07-A12 and B01-B06) as reported in the graph in Figure 2a in the manuscript.

**Well ID**	**[T_{11}] (nM) added to 10 nM of S_1-L**	k_f **(M^{-1} s^{-1})**	**Mean** k_f **(M^{-1} s^{-1})**	**SEM**
A02	2	1.45×10^5		
A03	4	1.26×10^5		
A04	6	1.15×10^5		
A05	8	1.15×10^5		
A06	10	1.15×10^5		
A08	2	1.33×10^5		
A09	4	1.19×10^5		
A10	6	1.14×10^5	1.20×10^5	2.69×10^3
A11	8	1.12×10^5		
A12	10	1.13×10^5		
B02	2	1.37×10^5		
B03	4	1.16×10^5		
B04	6	1.15×10^5		
B05	8	1.11×10^5		
B06	10	1.13×10^5		

Table S34. Observed second-order rate constants (k_f) for each trace of the 3 sets of replicas (E02-E06, E07-E12 and F01-F06) as reported in the graph in Figure 2b in the manuscript.

**Well ID**	**[R$_1$] (nM) added to 10 nM of S$_1$-T$_{11}$**	k_f **(M^{-1} s^{-1})**	**Mean** k_f **(M^{-1} s^{-1})**	**SEM**
E02	2	1.23×10^5		
E03	4	9.96×10^4		
E04	6	9.93×10^4		
E05	8	1.02×10^5		
E06	10	1.02×10^5		
E08	2	1.19×10^5		
E09	4	1.06×10^5		
E10	6	1.01×10^5	1.04×10^5	2.00×10^3
E11	8	9.96×10^4		
E12	10	9.69×10^4		
F02	2	1.02×10^5		
F03	4	1.09×10^5		
F04	6	9.75×10^4		
F05	8	1.02×10^5		
F06	10	9.69×10^4		

Table S35. Observed second-order rate constants (k_f) for each trace of the 3 sets of replicas (B07-B12, C01-C06 and C07-C12) as reported in the graph in Figure 2c in the manuscript.

**Well ID**	**[D^7] (nM) in which 10 nM of S_1-R_1-T_{11} are added**	k_f **(M^{-1} s^{-1})**	**Mean** k_f **(M^{-1} s^{-1})**	**SEM**
B08	2	7.94×10^4		
B09	4	6.96×10^4		
B10	6	7.23×10^4		
B11	8	7.47×10^4		
B12	10	7.82×10^4		
C02	2	7.43×10^4		
C03	4	6.80×10^4		
C04	6	6.71×10^4	7.36×10^4	1.22×10^3
C05	8	7.25×10^4		
C06	10	7.47×10^4		
C08	2	8.08×10^4		
C09	4	6.84×10^4		
C10	6	6.89×10^4		
C11	8	7.43×10^4		
C12	10	8.14×10^4		

SI.5.1.4 Reversibility of step 1

Data from a single replica, probing the reversibility of step 1 is shown in Figure S5. From the graph we observe negligible differences relative to the 0 nM control, suggesting that the reaction is functionally irreversible. The jumps in the fluorescence signal were due to the change of mechanical environment during injection and applying the seal on the plate.

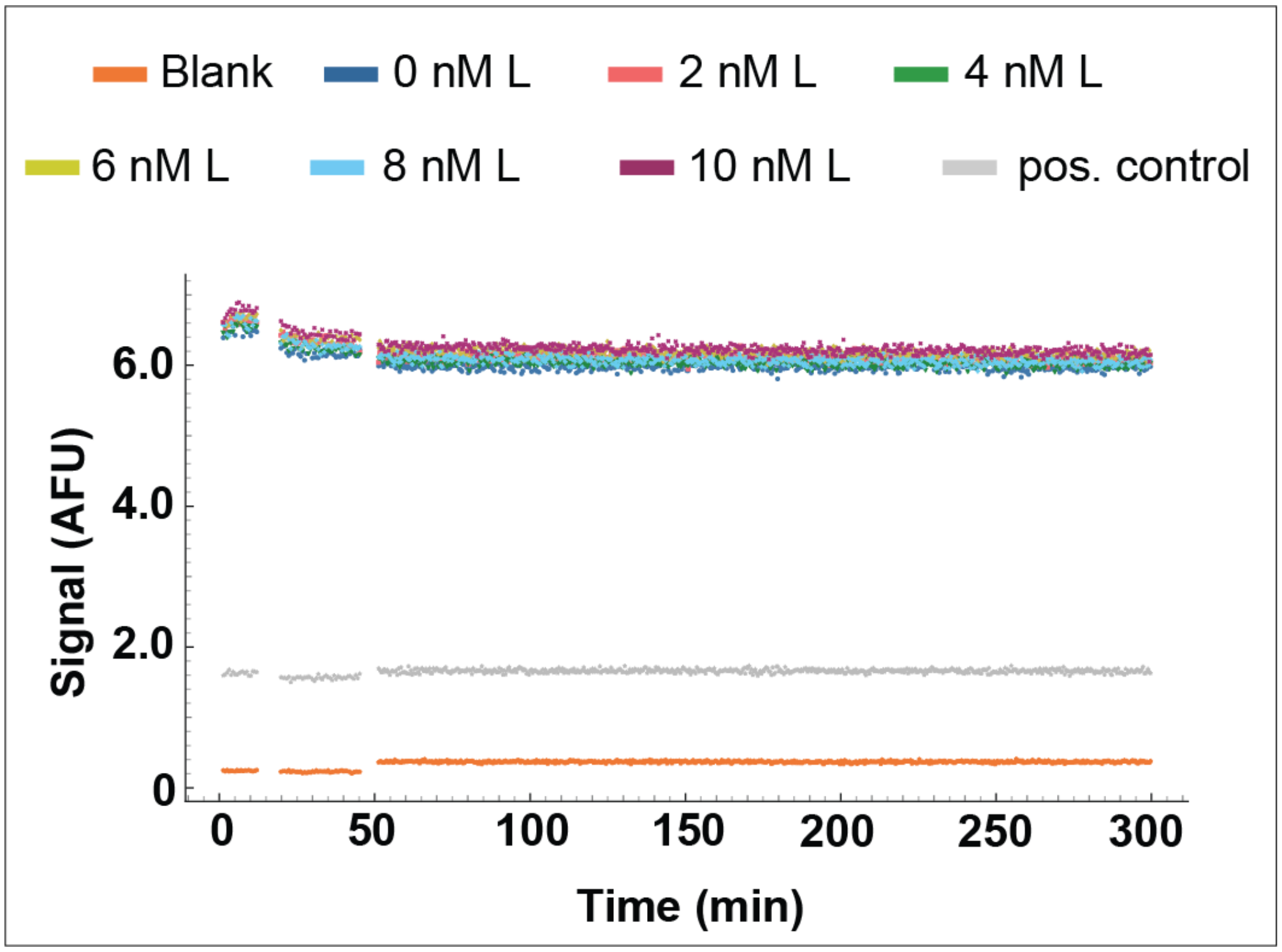

Figure S5. Reversibility test for step 1. Reactions were conducted according to the experimental protocol outlined in Table S15, with L added to a solution of S_1-T_{11}. The fluorescence in the ATTO 488 channel is plotted against time, with the 10 nM of S_1-L positive control experiment reported.

SI.5.1.5 Reversibility of step 3

Data from single replicas, probing the reversibility of step 3 is shown in Figure S6 and S7. From the graphs we observe negligible differences relative to the 0 nM control, suggesting that the reaction is functionally irreversible. The jumps in the fluorescence signal were due to the change of mechanical environment during injection and applying the seal on the plate.

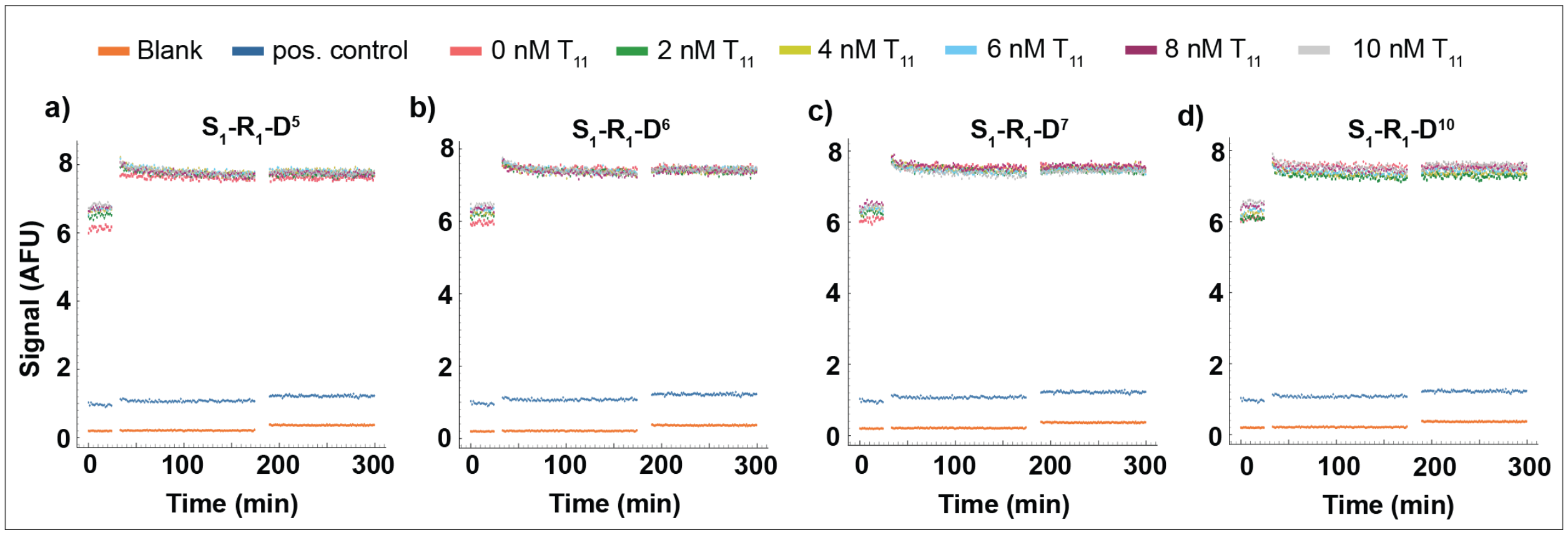

Figure S6. Reversibility test for step 3 for varying internal toeholds. Reactions were conducted according to the experimental protocol outlined in Table S16, with template T_{11} added to S_1-R_1-D^j, (j = 5, 6, 7, 10). The fluorescence in the ATTO 488 channel is plotted against time, with the 10 nM of S_1-R_1-T_{11} positive control experiment reported for comparison. No reaction was observed.

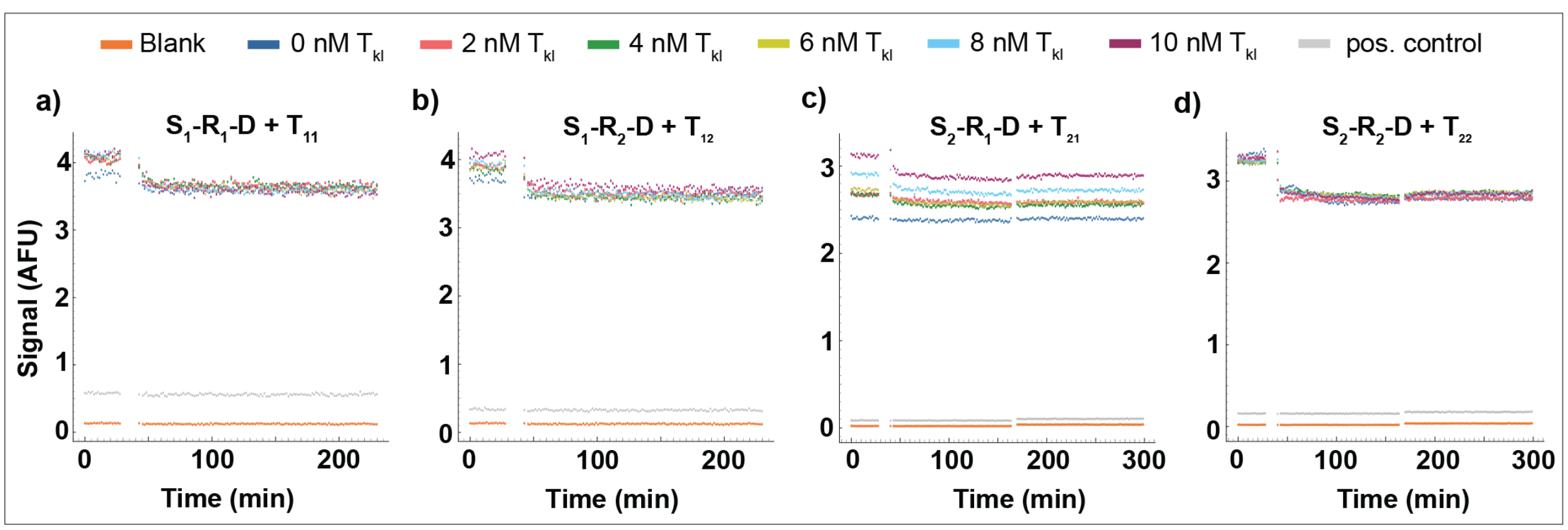

Figure S7. Reversibility test for step 3 for all orthogonal products. Reactions were conducted according to the experimental protocol outlined in Table S16, with template T_{kl} added to S_k-R_l-D^7. The fluorescence in the ATTO 488 channel (a-b) and Alexa Fluor 546 channel (c-d) are plotted against time, with the 10 nM of S_k-R_l-T_{kl} positive control experiment reported for comparison. No reaction was observed.

SI.5.2 Optimization of catalytic templating turnover

SI.5.2.1 Optimization of the toehold and the handhold lengths

Data from single replicas, probing catalytic turnover for different toehold and handhold lengths for an internal toehold of length 7 is plotted in Figure S8. We observe that while most systems show evidence of catalytic turnover, the template with toehold 7 and handhold 8 was the most efficient. From this comparative data, we observed that making the recognition domains longer could potentially slow down the overall turnover, presumably due to product inhibition.

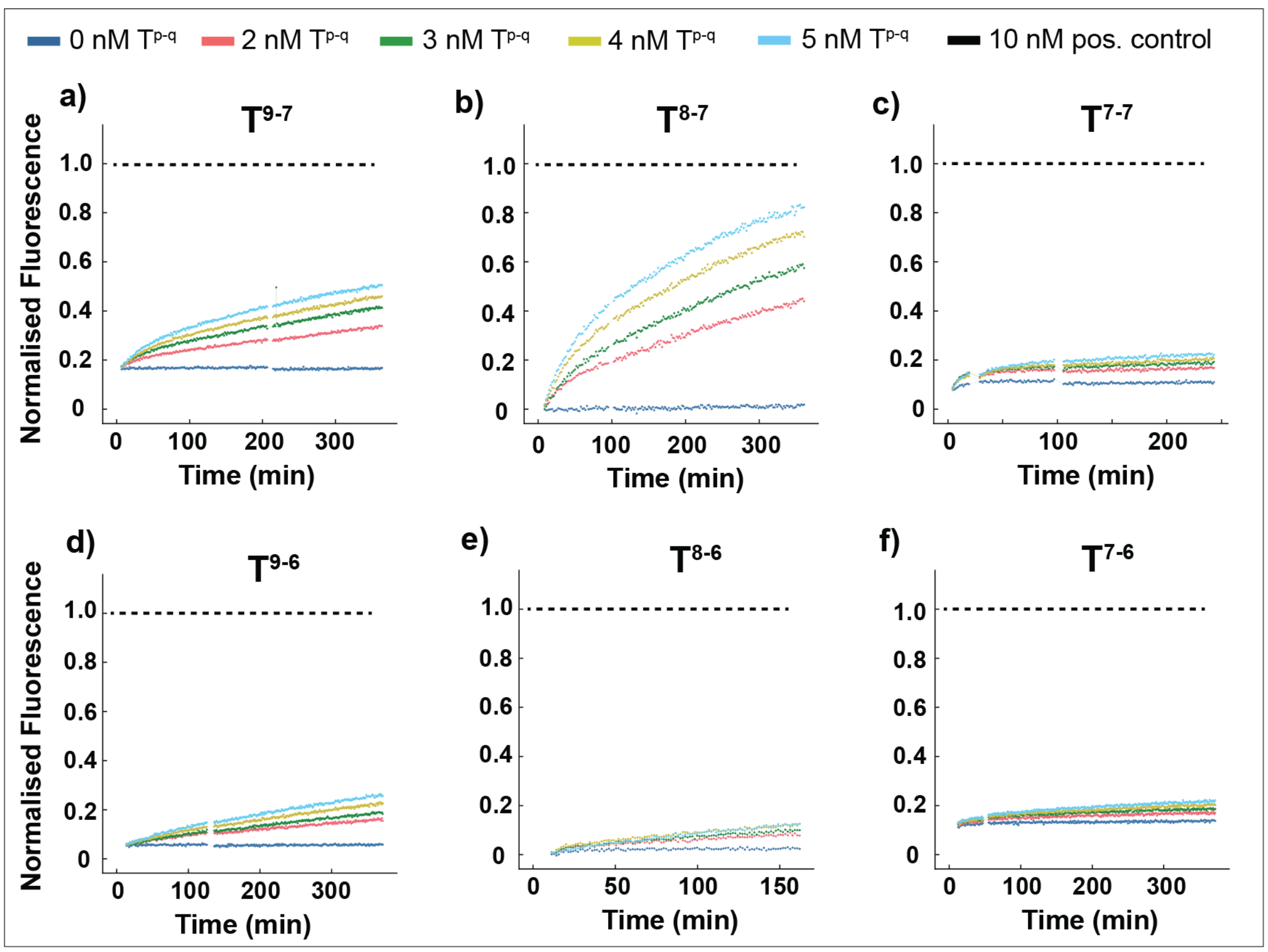

Figure S8. Catalytic turnover with variable lengths of toeholds and handholds. Reactions were conducted according to the experimental protocol outlined in Table S17, with template $T^{p\text{-}q}$ added to a solution of S_1-L, R_1 and D. The FRET in the ATTO 488-Alexa Fluor 546 (FRET) channel is plotted against time, with the 10 nM of S_1-R_1-D^7 positive control experiment reported for comparison. The normalised data are obtained by following the procedure in section SI.3.2.

Table S36. Fit parameters for catalytic turnover with different fuels.

Fuel	Rate constant k (min^{-1})	Michaelis constant K (nM)
D^5	0.000 108 87	–
D^6	0.000 134 03	–
D^7	0.009 329 95	7.61
D^{10}	0.000 760 03	–

SI.5.3 Catalytic templating regulated by excess of fuel molecule

In addition to the data in Figure 3e in the manuscript, we collected further data from three replicas for varying concentrations of D^7 and D^6, with the mean and range plotted in Figure S9.

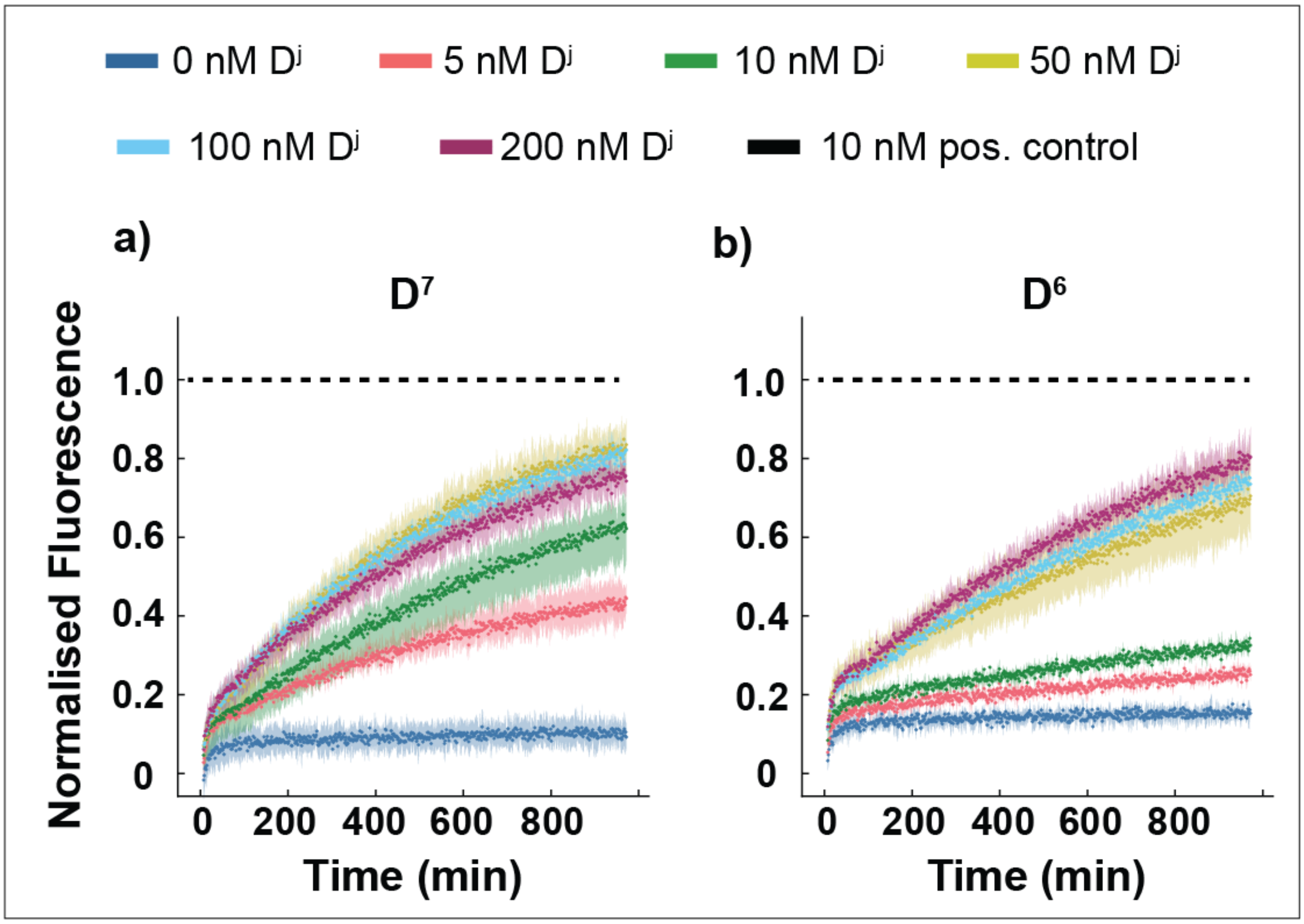

Figure S9. Catalytic turnover with varying concentration of fuels: (a) D^7; and (b) D^6. Reactions were conducted according to the experimental protocol outlined in Table S19, with template T_{11} added to S_l-L, R_1 and D^7/D^6. The fluorescence in the ATTO 488 channel, normalized with respect 10 nM of S_1-R_1-$D^{6/7}$ according to the procedure in section SI.3.2, is plotted against time.

SI.5.4 Catalytic templating with high monomer-to-template ratio

In addition to the data in Figure 3f in the manuscript, we collected a third replica which showed anomalous behaviour (most likely due to formation of a bubble) in one of the traces (shown in light blue). For completeness, we report that replica in Figure S10a. In Figure S10b we show the combined data for all three replicas.

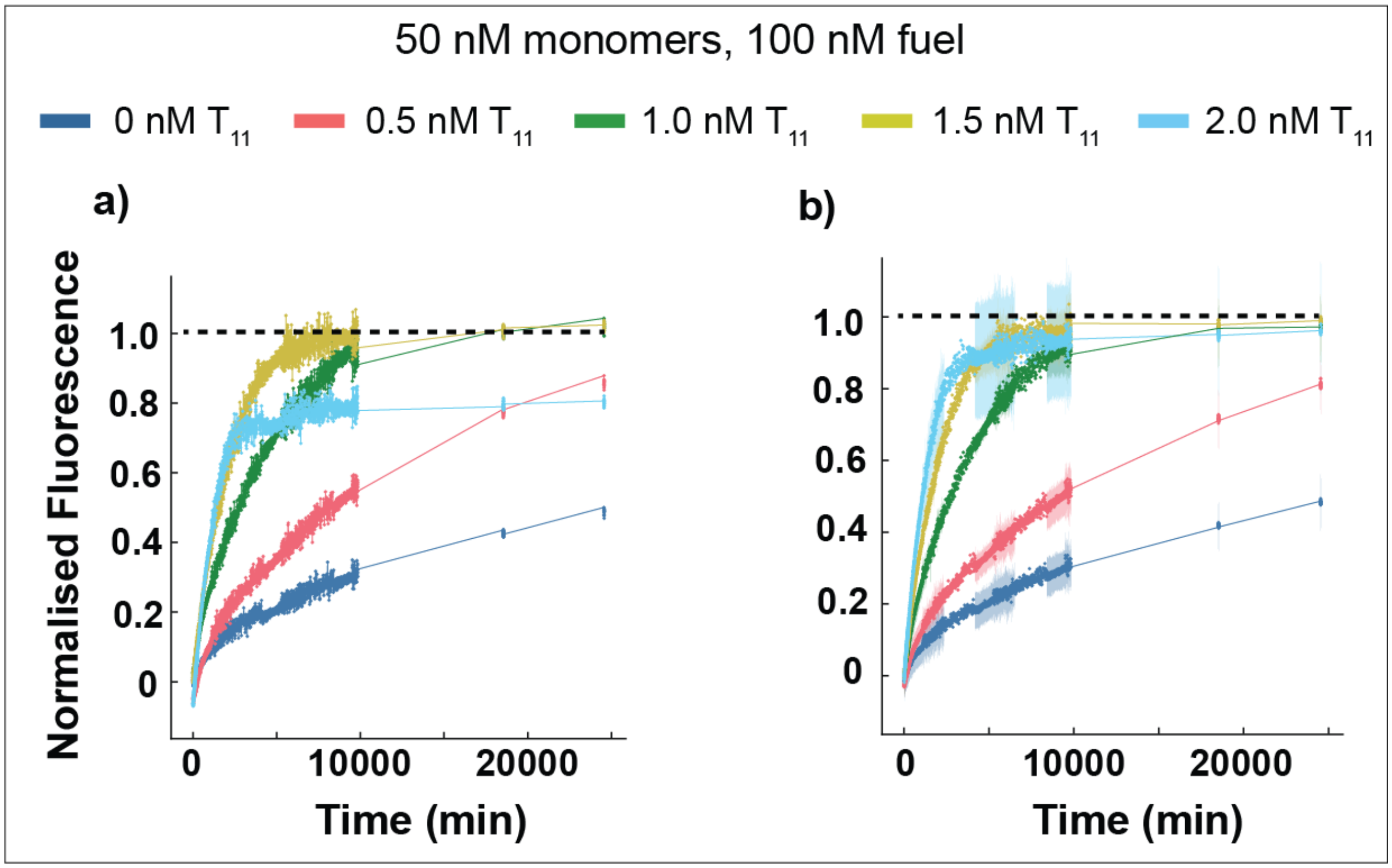

Figure S10. Catalytic templating with high monomer to template concentration ratios. Reactions were conducted according to the experimental protocol outlined in Table S21, with template T_{11} added to S_l-L, R_1 and D^7. The fluorescence in the ATTO 488 channel, normalized with respect 10 nM of S_1-R_1-D^7 according to the procedure in section SI.3.2, is plotted against time. In (a), we show a single replica with anomalous behaviour in the 2.0 nM trace, and in (b) we combine that data with that in Figure 3f, also including subsequent measurements taken at long time.

SI.5.5 Reactions for orthogonal dimerization

SI.5.5.1 Full reaction for orthogonal set 2

Data from a single replica, showing that the dimerisation of the second set of monomers S_2 and R_2 can be effectively templated by T_{22} is plotted in Figure S11.

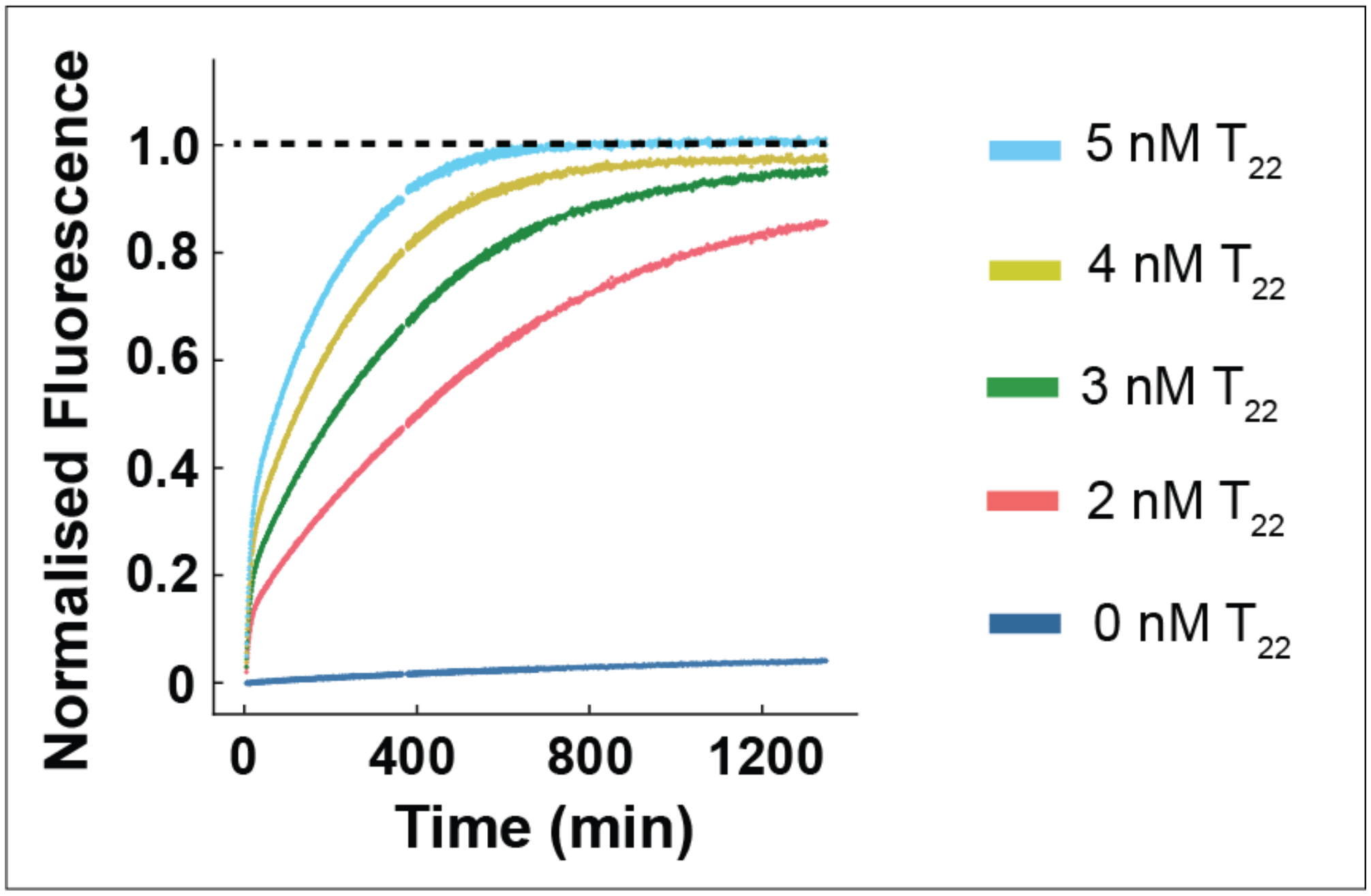

Figure S11. Templating reactions of the second orthogonal set showing catalytic turnover. Reactions were conducted according to the experimental protocol outlined in Table S22, with template T_{22} added to S_2-L, R_2 and D^7. The fluorescence in the Alexa Fluor 546 channel, normalized with respect 10 nM of S_2-R_2-D^7 according to the procedure in section SI.3.2, is plotted against time.

SI.5.5.2 Dimerization reactions of each pair of monomers triggered with four different templates

Data complementary to that in Figure 4a in the manuscript showing the behaviour of the other three pairs of monomers, when challenged with 4 different templates, is plotted in Figure S12. All datasets are obtained from single replicas.

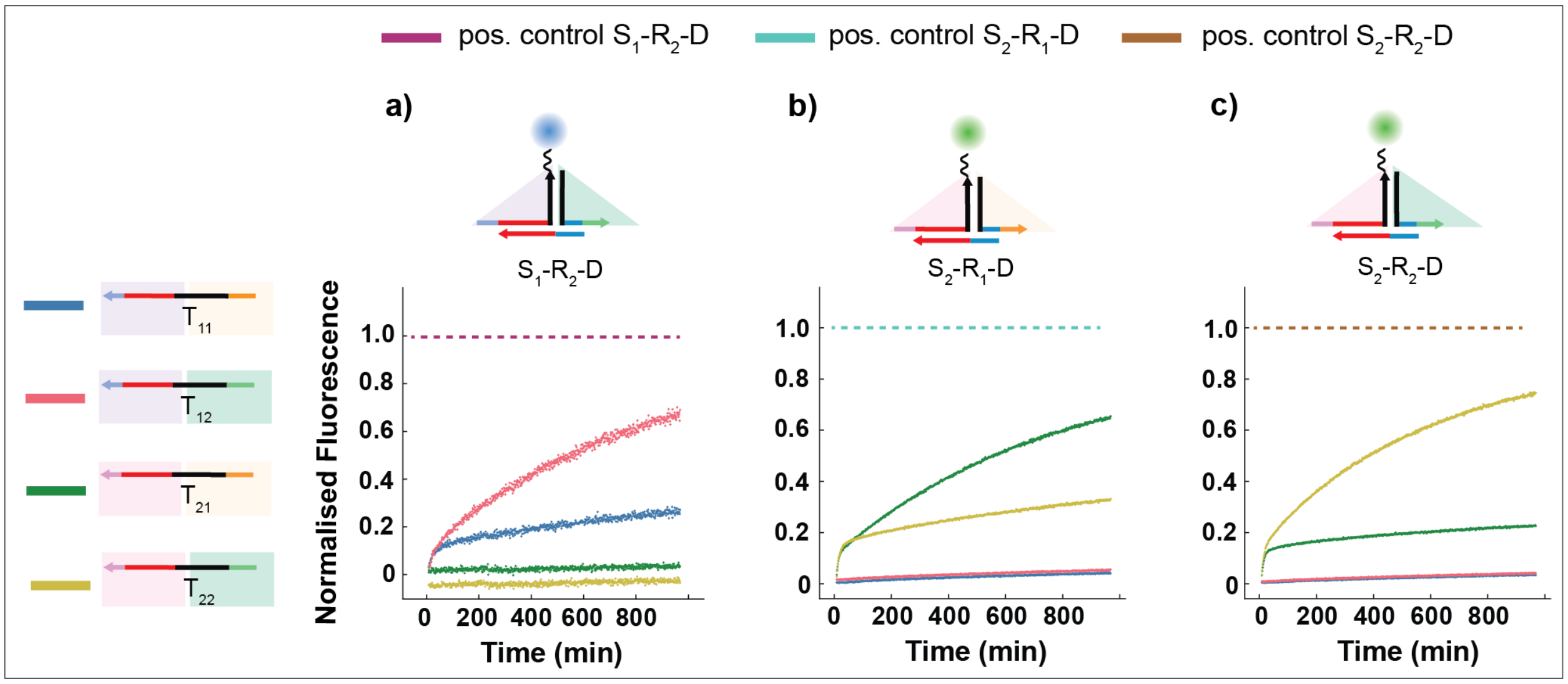

Figure S12. Specificity test for the orthogonal templates with each pair of monomers. Reactions were conducted according to the experimental protocol outlined in Tables S23, with templates T_{ij} separately added to solutions of: S_1-L, R_2 and D^7 in (a); S_2-L, R_1 and D^7 in (b); and S_2-L, R_2 and D^7 in (c). The fluorescence in the ATTO 488 channel (in (a)) or Alexa Fluor 546 channel (in (b) and (c)), normalized with respect 10 nM of the relevant product S_k-R_l-D^7 according to the procedure in section SI.3.2, is plotted against time.

SI.5.5.3 Specificity of the templates in a pool of monomers

In Figure S13, we plot for completeness the full gel image corresponding to Figure 4e in the manuscript, following the reaction protocol as described in Table S25.

To calculate the % yield of each of the products, as reported in Table S37 we used the individual 16 bit images, and not the composite images, for estimating the yields of the concerned products. For this, the bands for the pre-formed products (positive controls), were used as the references.

First, the backgrounds were subtracted from images to obtain sharp bands. Then the reference positive control band was selected to calculate the integrated pixel density, and was treated as the reference value for 100%. Then the integrated pixel densities were also calculated for the concerned bands from the other lanes, and were compared against the reference values to estimate the yields of the products. The image from the Cy2 channel was used to estimate the yields of products containing S_1, and the image from the Cy3 channel was used for products containing S_2.

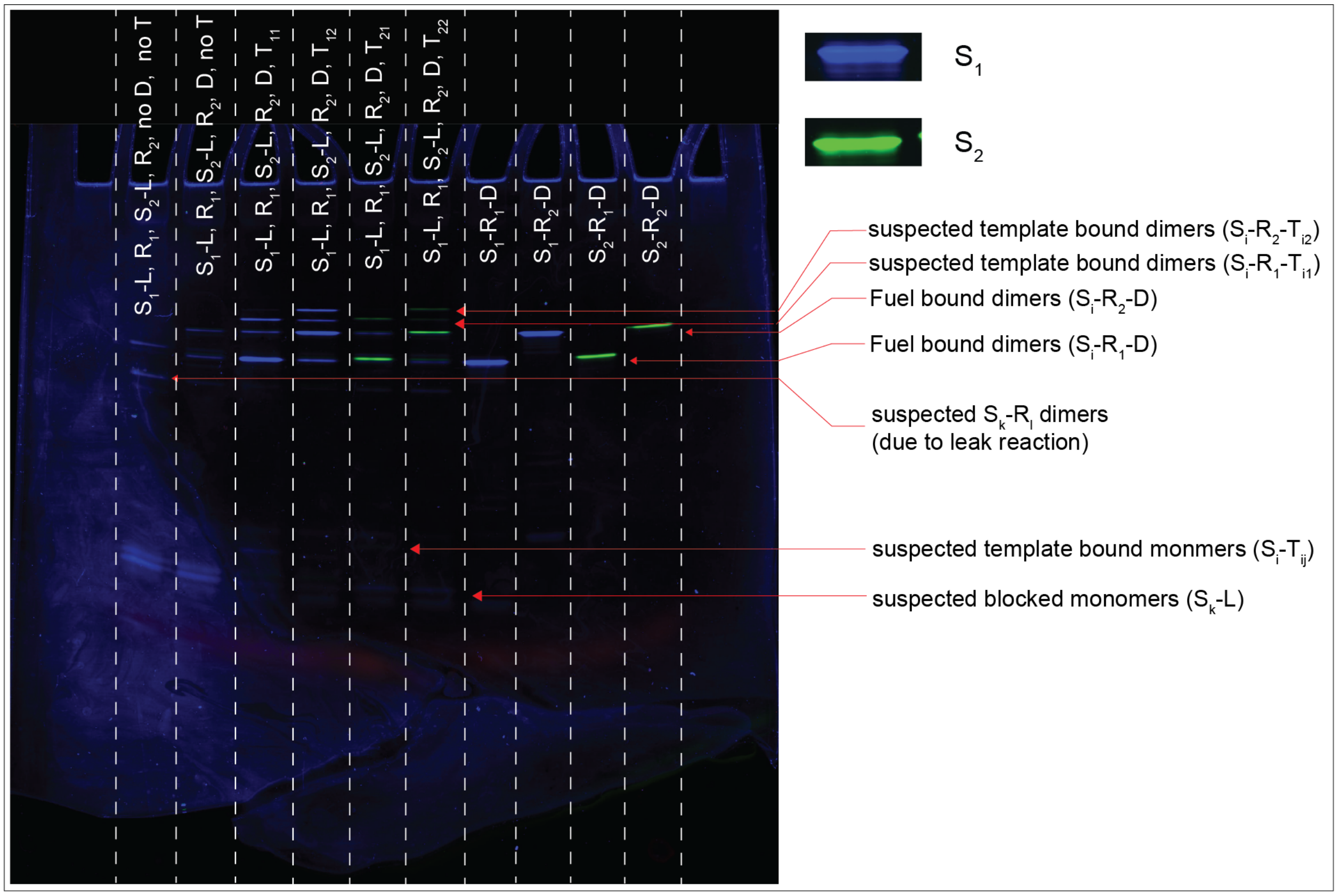

Figure S13. Gel electrophoresis demonstrating specificity of templates and orthogonal dimerization. Reactions were conducted according to the experimental protocol outlined in Table S25, with one of four different templates added to a mixture of all four monomers and fuel D.

Table S37. Percentage yield of the products in the respective lanes with respect to the positive controls.

	S_1–R_1–D	S_1–R_2–D	S_2–R_1–D	S_2–R_2–D
Controls	100	100	100	100
Lane 6	5.0965	3.0354	14.3944	49.6285
Lane 5	3.1786	3.7226	60.9613	9.4200
Lane 4	10.6144	55.9954	8.6086	6.7713
Lane 3	63.1970	7.3834	8.0688	8.0237
Lane 2	7.3172	6.1921	9.5497	8.1650

SI.5.5.4 Gel showing non-reactivity of second monomer and fuel prior reaction

Reaction mixtures were prepared as reported in Figure S14. The reaction mixtures were incubated at 4 °C for 48 h. Then the reaction mixtures and controls were loaded in a PAGE following the protocol in the gel electrophoresis section of the methods in the main manuscript. In this case, the gel was run for 75 min and then imaged on gel scanner. Following this procedure, the gel was stained with SYBR Gold dye and imaged again.

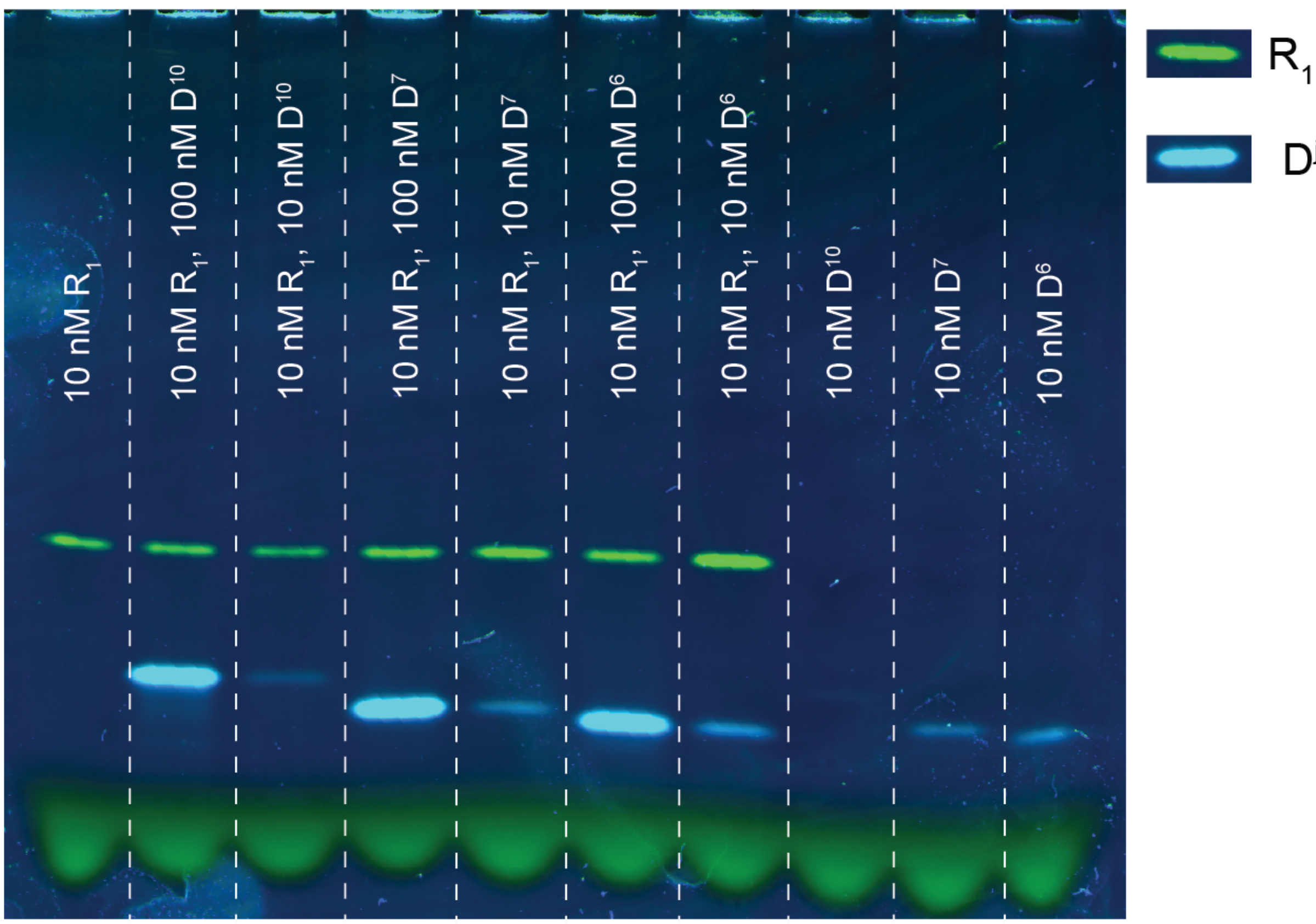

Figure S14. Gel electrophoresis demonstrating no reaction between second monomer and fuel before the triggering the reaction.

SI.5.5.5 Catalytic templating reactions with variable concentrations of inputs

Data from triplicate reactions for each, showing the effect of varying input concentration on catalytic templating reactions controlled by upstream DNA circuitry is plotted in Figure S15.

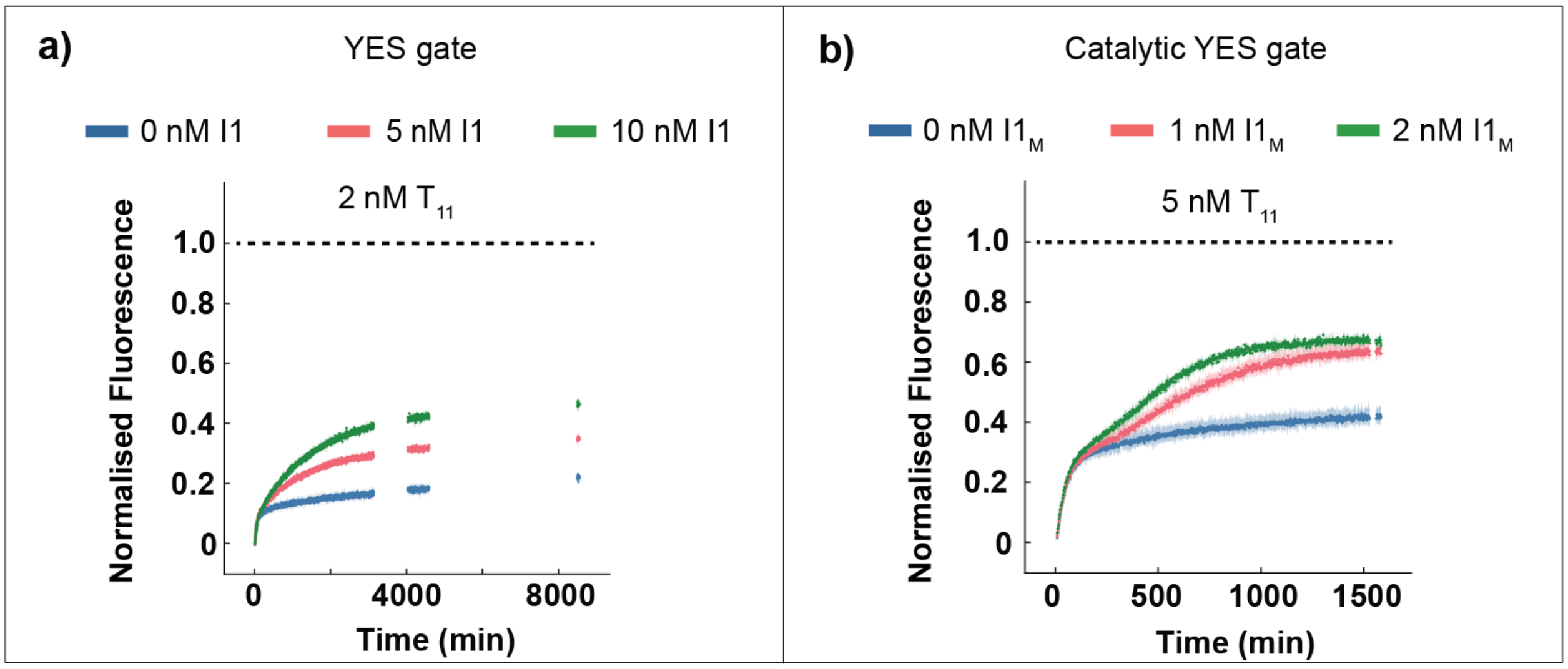

Figure S15. Catalytic templating coupled with (a) YES gate and (b) catalytic YES gate with varying concentrations of the input. Reactions were conducted according to the experimental protocols outlined in Tables S31 and S32.

SI.5.5.6 Schematic of a trimerization reaction network design following the proposed mechanism

We propose an extension to our system to exhibit a catalytic templated trimerization reaction as shown in Figure S16. In this design the template has three recognition sites *th*, *rd* and *hh* for monomer binding. Terminal monomers S_1 and P_1 are mechanistically similar to the monomeres used in the dimerization reaction. The second monomer R_1 now has two binding interfaces. the one on the left binds to S_1 and the one in the right binds to P_1. We employ an universal fuel D which displaces the template from the product. Note that the reactions need not proceed in exactly the order shown; we anticipate that fuel concentration can be tuned to optimize the fraction of reactions that lead to fully-formed products rather than truncated variants.

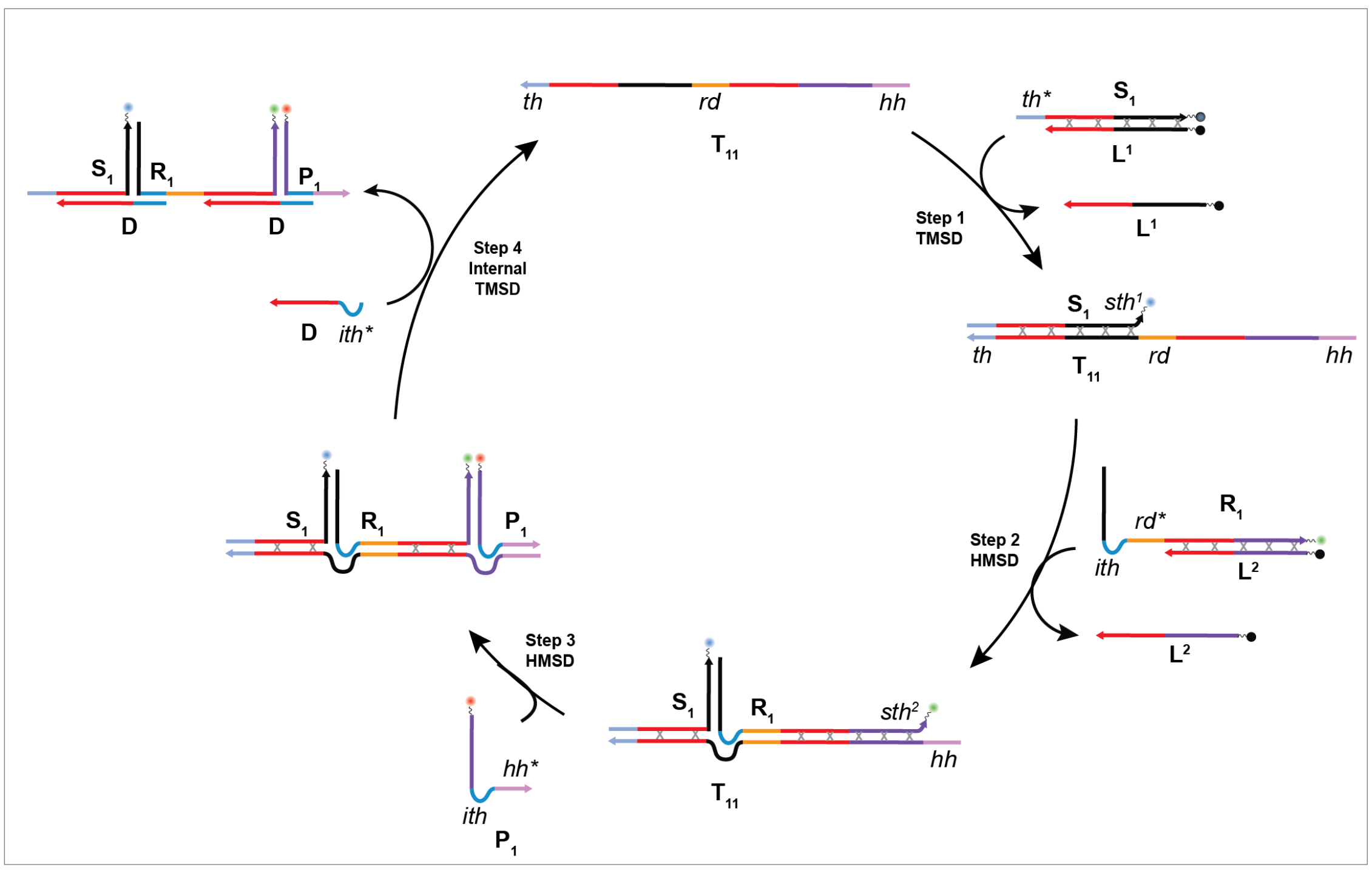

Figure S16. Schematics of a trimerization reaction network design following the proposed mechanism.
